# Prevalence, Characteristics, and Genetic Architecture of Avoidant/Restrictive Food Intake Phenotypes

**DOI:** 10.1001/jamapediatrics.2025.4786

**Published:** 2025-11-24

**Authors:** Ludvig Daae Bjørndal, Elizabeth C. Corfield, Laurie J. Hannigan, Ziada Ayorech, Cynthia M. Bulik, Hunna J. Watson, Lisa Dinkler, Samuel J. R. A. Chawner, Stefan Johansson, Ole A. Andreassen, Helga Ask, Alexandra Havdahl

**Affiliations:** 1 PROMENTA Research Center, Department of Psychology, University of Oslo, Oslo, Norway; 2 Psychiatric Genetic Epidemiology Group, Research Department, Lovisenberg Diaconal Hospital, Oslo, Norway; 3 PsychGen Centre for Genetic Epidemiology and Mental Health, Norwegian Institute of Public Health, Oslo, Norway; 4 MRC (Medical Research Council) Integrative Epidemiology Unit, Population Health Sciences, Bristol Medical School, University of Bristol, Bristol, United Kingdom; 5 Department of Medical Epidemiology and Biostatistics, Karolinska Institutet, Stockholm, Sweden; 6 Department of Psychiatry, School of Medicine, University of North Carolina at Chapel Hill, Chapel Hill; 7 Department of Nutrition, University of North Carolina at Chapel Hill, Chapel Hill; 8 Discipline of Psychology, School of Population Health, Curtin University, Perth, Western Australia, Australia; 9 Department of Psychiatry, School of Medicine, University of North Carolina at Chapel Hill, Chapel Hill; 10 Division of Paediatrics, School of Medicine, University of Western Australia, Perth, Western Australia, Australia; 11 Centre for Neuropsychiatric Genetics and Genomics, Division of Psychological Medicine and Clinical Neurosciences, Cardiff University, Cardiff, United Kingdom; 12 Mohn Center for Diabetes Precision Medicine, Department of Clinical Science, University of Bergen, Bergen, Norway; 13 Department of Paediatrics and Adolescent Medicine, Haukeland University Hospital, Bergen, Norway; 14 Division of Mental Health and Addiction, Oslo University Hospital, Oslo, Norway; 15 Institute of Clinical Medicine, University of Oslo, Oslo, Norway

## Abstract

**Importance:**

A narrow range of food consumption and/or restricted eating is a core feature of avoidant/restrictive food intake (ARFI) disorder. However, there is limited knowledge of developmental characteristics of children with ARFI and its etiological influences, which constrains research, prevention, and intervention efforts.

**Objective:**

To estimate the prevalence of ARFI phenotypes in a population-based sample, examine developmental characteristics across childhood, and investigate the genetic architecture of ARFI using genome-wide association analyses.

**Design, Setting, and Participants:**

This preregistered study used data from children born from 1999 to 2009 in the population-based Norwegian Mother, Father, and Child Cohort Study (MoBa), with mother-reported data on ARFI symptoms at 3 and 8 years and linkage with diagnostic data from population health registries. Data were analyzed from March 2024 to May 2025.

**Exposures:**

Multiple items were used to identify children with broad ARFI. These children were subclassified into 3 groups based on symptom persistence: ARFI–broad transient (only at age 3 years), emergent (only at age 8 years), and persistent (ages 3 and 8 years). Children in these groups with 1 or more indicators of clinical significance (eg, nutritional deficiency) were further classified into ARFI-clinical subgroups.

**Main Outcomes and Measures:**

ARFI groups were compared across developmental characteristics from 6 months to 14 years. Genome-wide methods were used to examine single-nucleotide variant (SNV) heritability (SNV-*h*^2^), conduct genetic association analyses, and quantify genetic correlations with other phenotypes.

**Results:**

Of 35 751 children with available ARFI assessments at 3 and 8 years (18 236 male [51%]), the prevalence of ARFI–broad persistent, transient, and emergent was 2129 (6.0%), 6338 (17.7%), and 3001 (8.4%), respectively. The prevalence of ARFI-clinical persistent, transient, and emergent was 624 (1.8%), 1157 (3.2%), and 484 (1.4%), respectively (2265 [6.3%] overall). Children with ARFI–broad persistent exhibited more developmental difficulties compared with children with no ARFI. SNV-*h*^2^ ranged from 8% to 16%. Two independent genome-wide significant loci were identified. For ARFI-clinical, a significant association was identified with *ADCY3* (*z* = 5.42; *P* = 3.03 × 10^−8^). Small to moderate genetic correlations were observed for ARFI-broad, ARFI-clinical and mental health, cognitive/educational, anthropometric, food-associated, and gastrointestinal disorder phenotypes.

**Conclusions and Relevance:**

This cohort study found that the prevalence of ARFI in the general pediatric population was substantial, and affected children had an associated elevated risk of developmental difficulties across multiple domains. Findings suggest a need for broad support interventions and advance understanding of the genetic underpinnings of ARFI.

## Key Points

**Question:**

What is the prevalence of avoidant/restrictive food intake (ARFI) in the general pediatric population, developmental characteristics of affected children, and the genetic underpinnings of ARFI symptoms?

**Findings:**

In this cohort study of 35 751 children, those with ARFI exhibited more developmental difficulties compared with children with no ARFI. Two independent genome-wide significant loci and an association with *ADCY3* were identified, and small to moderate genetic correlations were observed between ARFI and mental health, cognitive, anthropometric, food-associated, and gastrointestinal phenotypes.

**Meaning:**

This study suggests that the prevalence of ARFI was considerable and affected children had an associated elevated risk for developmental difficulties.

## Teaser

This cohort study investigates the prevalence of avoidant/restrictive food intake (ARFI) in the general pediatric population, developmental characteristics of affected children, and the genetic underpinnings of ARFI symptoms.

## Introduction

Many children experience eating and feeding challenges.^1^ A common dysregulated eating pattern involves food avoidance and restriction, which may be linked to heightened sensitivity to sensory characteristics of food, limited food interest, or concerns about potentially adverse effects of eating, including fear of discomfort or choking. The prevalence of broad eating difficulties such as picky eating is considerable: 10% to 15% for picky eating at different ages in early childhood^2^ and 5.5% for persistent picky eating across childhood.^3^

Avoidant/restrictive food intake disorder (ARFID), included as a diagnosis in the *Diagnostic and Statistical Manual of Mental Disorders* (Fifth Edition; *DSM-5*)^4^ and the *International Classification of Diseases, 11th Revision*,^5^ is characterized by a limited range of foods consumed and/or restricted food intake, resulting in nutritional deficiencies or failure to meet energy needs. ARFID was incorporated in the *DSM-5* to address the need for a diagnostic category for individuals with persistent and clinically significant avoidant/restrictive eating not primarily motivated by concerns about weight or body image.^6^

Understanding the prevalence of ARFID and associated symptoms in the population is essential for guiding public health policies. Prevalence estimates range from 1.5% to 64%, varying by sample type (clinical vs community), age, and ARFID definition.^7,8,9^ A recent meta-analysis^10^ of 26 nonclinical studies estimated the prevalence of ARFID at 4.5%, with studies characterized by mostly small, cross-sectional samples and heterogeneity in age range and ARFID measures. Little is known about the emergence and persistence of ARFID and associated phenotypes like picky eating in childhood.^2^ Furthermore, most studies have been conducted in North America, and the generalizability of findings to other geographical contexts is unclear.^9^

Children with ARFID symptoms exhibit higher levels of somatic complaints and emotional, attention, and social problems,^11,12^ and persistent picky eating has been linked to developmental difficulties.^3^ ARFID commonly co-occurs with symptoms and diagnoses of neurodevelopmental (eg, autism), mental health (eg, anxiety), and gastrointestinal conditions (eg, irritable bowel syndrome).^9,13,14,15^ However, longitudinal studies in general population samples are needed to better understand how ARFID symptoms evolve across development and associations with clinical impairment (eg, malnutrition, growth deficits). This knowledge may inform prevention and intervention efforts to support children experiencing ARFI.

Several eating-associated phenotypes in childhood exhibit substantial heritability, including food fussiness,^16,17,18^ a predilection for fruits and vegetables,^16,19^ and appetitive characteristics.^20^ A recent twin study^21^ estimated ARFID heritability at 79%, indicative of a strong genetic component to ARFID risk. This aligns with high twin-based heritability estimates for eating disorders more generally.^22^ The scarcity of studies examining genetic underpinnings of ARFID is a key gap in the literature.^23^ Enhancing knowledge of genetic factors associated with ARFI could improve understanding of its etiological influences.

Here, we estimate the prevalence of ARFI phenotypes in children aged 3 and 8 years in the Norwegian Mother, Father, and Child Cohort Study (MoBa). These phenotypes were constructed to identify children with food intake patterns characterized by avoidance and/or restriction both with and without apparent clinical impairment. We examine developmental characteristics from early childhood (6 months) until adolescence (14 years). We use genome-wide methods to quantify the single-nucleotide variant (SNV) heritability of ARFI phenotypes, identify associated genetic variants, and quantify genetic correlations with other eating-associated, neurodevelopmental, mental health, neurological, cognitive, growth, and gastrointestinal phenotypes. Our hypotheses were as follows: (1) the prevalence of ARFI phenotypes ranges between 2% and 10%, (2) children with ARFI exhibit early-onset and persistent difficulties across developmental domains, (3) common genetic variants explain a substantial proportion of individual differences in ARFI, and (4) there is genetic overlap between ARFI, neuropsychiatric conditions, medical conditions, and other traits.

## Methods

The establishment of the MoBa study and initial data collection were based on a license from the Norwegian Data Protection Agency and approval from The Regional Committees for Medical and Health Research Ethics. The MoBa cohort is currently regulated by the Norwegian Health Registry Act. This study was approved by The Regional Committees for Medical and Health Research Ethics (2016/1702). This study is reported according to the Strengthening the Reporting of Observational Studies in Epidemiology (STROBE) reporting guidelines.^24^

### Participants

We used data from children who are part of the MoBa study,^25^ a population-based pregnancy cohort study conducted by the Norwegian Institute of Public Health. Participants were recruited from all over Norway from 1999 to 2008. Invitations to participate were sent to women, and invited individuals provided written, informed consent to participate. The MoBa study includes approximately 114 500 children, 95 200 mothers, and 75 200 fathers. Genotype data are available for approximately 80% of the cohort.^26^ Blood samples were obtained from both parents during pregnancy and from mothers and children (umbilical cord) at birth.^27^ Data on participant race and ethnicity were not gathered.

Data from the MoBa study can be linked with national registries in Norway^28^ to further assess history of eating disorder diagnosis and clinical indicators. We used data from the Norwegian Control and Payment of Health Reimbursements Database, which includes records of interactions with primary health care services (2008-2021), with diagnostic codes in accordance with the *International Classification of Primary Care*, *Second Revision *(*ICPC-2*). We also used data from the Norwegian Patient Registry (NPR), which includes records of interactions with specialist health care services (2008-2023), including diagnostic codes in accordance with the *International Statistical Classification of Diseases and Related Health Problems*,* Tenth Revision *(*ICD-10*).

### Operationalization of ARFI Phenotypes

We defined 2 phenotypes of interest: 1 broader category of ARFI (ARFI-broad) and 1 additionally including clinical indicators (ARFI-clinical). These phenotypes were constructed to capture the key feature of ARFID diagnosis: symptom(s) of ARFI. Furthermore, we aimed to capture a broad range of presentations, including children both with and without various indicators of clinical significance. However, some ARFID diagnostic criteria could not be directly assessed in our data (ie, eating disturbance not explained by lack of food, culturally sanctioned practices, or body weight or shape concerns).

#### ARFI-Broad

Children were classified with ARFI-broad at 3 and/or 8 years if they had 1 or more mother-reported symptoms of ARFI at these ages (all symptoms reported in eTable 1 in Supplement 1). MoBa study questionnaires for other ages contained too few or nonspecific items to assess ARFI. The highest response category (ie, very true or often true, totally agree, or always) was used as the threshold for identifying the presence of a symptom. ARFI-broad was further categorized into 3 patterns of symptoms: persistent (present at both ages 3 and 8 years), transient (present only at age 3 years), and emergent (present only at age 8 years).

#### ARFI-Clinical

ARFI with clinically significant consequences (ARFI-clinical) was defined as meeting the criteria for ARFI-broad (persistent, transient, and/or emergent) and at least 1 indicator of clinical significance plausibly associated with ARFI, identified using other MoBa items or diagnostic codes from health registries (Table 1).

**Table 1.** Indicators of Clinical Significance in Avoidant/Restrictive Food Intake (ARFI)–Clinical

Indicator	Diagnostic codes/items
**With weight loss or failure to gain weight/grow**	
No weight gain or underweight for >1 y	MoBa items^a^
	T08^b^
BMI <5th percentile	MoBa items^c^
Delayed physical growth	T10^b^
Abnormal weight loss	R63.4^d^
Unspecified severe protein-energy malnutrition	E43^d^
Delayed physical development due to protein-calorie malnutrition	E45^d^
**With nutritional deficiency**	
Child poor feeding preparation/management, nutrition problem, malnutrition	T91^b^, B81^b^, E40-E42^d^, E44^d^, E46^d^, E50^d^, E51^d^, E53^d^, E55^d^, E56^d^, E58-E61^d^, E63^d^, E64^d^
Vitamin deficiency or nutritional disturbance	
Malnutrition and other nutritional deficiencies	
Unspecified protein/energy malnutrition	
**With eating difficulties of clinical significance**	
Other ED (eg, psychogenic appetite loss)	F50.8^d^
Unspecified ED	F50.9^d^
ED in childhood (eating avoidance or extreme pickiness)	F98.2^d^
Lack of appetite (anorexia)	R63.0^d^
Difficulties with food intake	R63.3^d^
Insufficient intake of food and liquids	R63.6^d^
Reduced appetite	T03^b^
Child eating difficulties	T04^b^
ED in children (feeding problems)	P11^b^

Abbreviations: BMI, body mass index; ED, eating disorder; *ICD-10*, *International Statistical Classification of Diseases and Related Health Problems, Tenth Revision*; *ICPC-2*, *International Classification of Primary Care, Second Revision*; MoBa, Norwegian Mother, Father, and Child Cohort Study.

^a^ Assessed using MoBa item asking if the child has shown too little weight gain now or previously (at child age of 3 years).

^b^ *ICPC-2* diagnostic code.

^c^ Calculated using mother-reported MoBa items for child height and weight at 3 and 8 years (5th percentile used following the procedure of Dinkler et al^21^).

^d^ *ICD-10* diagnostic code. All diagnoses were required to have been first assigned between 2 and 10 years of age.

### Developmental Characteristics

Developmental characteristics were assessed using multiple measures administered in the MoBa^29^ study across 14 years of follow-up (all measures reported in the eMethods in Supplement 1) and include the following: eating-associated difficulties, language development, motor skills, social communication and prosocial behavior, restricted and repetitive behaviors, emotional and behavioral difficulties, and attention/hyperactivity difficulties. Lifetime diagnoses (neurodevelopmental, mental health, neurological, gastrointestinal) were identified from the NPR.

### Statistical Analysis

Children with ARFI–broad persistent and no ARFI-broad were compared for differences in characteristics and lifetime diagnosis prevalence using independent sample *t* tests and 2-proportion *z* tests with α = .05. ARFI–broad persistent was selected for this analysis as it likely reflects more sustained and clinically significant ARFI. We controlled for the false discovery rate (FDR) by correcting for 56 tests (ie, 56 characteristics) and 9 tests (ie, 9 lifetime diagnostic groups) using the Benjamini and Hochberg method.^30^

To maximize sample sizes, genetic analyses included all children with data at either age 3 or 8 years, in contrast to the main phenotypic analyses, which required data at both ages. Cases for genome-wide association studies (GWAS) of ARFI-broad included children with at least 1 ARFI symptom at (1) 3 years, (2) 8 years, (3) 3 or 8 years, and (4) both 3 and 8 years. Cases for GWAS of ARFI-clinical included children with at least 1 symptom at (1) 3 or 8 years and clinical significance indicator and (2) both 3 and 8 years and clinical significance indicator. Children with anorexia nervosa recorded in the NPR were excluded as cases in all analyses to minimize risk of misclassification. In sensitivity analyses, we additionally excluded cases with diagnoses of general medical conditions that could plausibly explain the dysregulated eating pattern. Controls comprised children with no ARFI-broad at any age and no eating disorder or relevant general medical condition diagnoses in NPR.

Quality-controlled genotype data were available for participants with European genetic ancestry.^26^ We examined the genetic architecture of ARFI-broad and ARFI-clinical by conducting genome-wide association studies (GWAS) using the C++ program REGENIE (Regeneron Genetics Center),^31^ which accounts for case-control imbalance and relatedness. Covariates included the child’s sex registered at birth and year of birth, genotyping batch, as well as the first 20 genetic principal components to adjust for population stratification.

The genome-wide significance threshold adjusted for multiple testing based on the European linkage disequilibrium (LD) structure (*P* < 5 × 10^−8^) was used. Identified genome-wide significant loci were investigated using conditional analyses accounting for LD structure, implemented in the GCTA software package (Yang Lab),^32,33^ to identify if multiple association signals exist in 1 locus. Additionally, regional plots were generated using LocusZoom (University of Michigan Center for Statistical Genetics)^34^ to visualize the recombination and LD patterns in the 400 Kb flanking region of the genome-wide significant loci.

We subsequently used the summary statistics to conduct gene-based association analyses using MAGMA (Complex Trait Genetics Lab).^35^ Gene-based association analyses used a Bonferroni corrected threshold of *P* < .05/number of tested genes.

We used summary statistics from the GWAS to estimate SNV heritability with LD score regression (LDSC), implementing the European genetic ancestry LD scores from the 1000 Genomes reference panel. Next, we used LDSC to examine genetic correlations between ARFI-broad, ARFI-clinical, and neurodevelopmental and mental health, neurological, cognitive and educational, growth, gastrointestinal, food predilection, and appetite- and satiety-associated hormone phenotypes (eTable 3 in Supplement 1). The threshold for statistical significance was a 2-sided *P* value < .05. All analyses were conducted from March 2024 to May 2025 using R, version 4.1.2 (R Project for Statistical Computing).^36^ The phenotools package, version 0.3.0, was used for preparing MoBa data.^37^ The study was preregistered,^38^ and an overview of deviations from the preregistered approach is provided in eTable 4 in Supplement 1.

## Results

### Prevalence of Avoidant/Restrictive Food Intake

Invitations to participate in the MoBa study were sent to 277 702 women, and 41% of invited individuals consented to participate. Among the 35 751 children (17 515 female [49%]; 18 236 male [51%]) with data at both age 3 and 8 years, 11 468 (32.1%) were classified with ARFI-broad (Table 2): 2129 (6.0%) with ARFI–broad persistent (ie, symptoms present at 3 and 8 years), 6338 (17.7%) with transient (ie, 3 years only), and 3001 (8.4%) with emergent (ie, 8 years only). A total of 24 283 children (67.92%) were classified with no ARFI-broad. Cases for GWAS of ARFI-broad included children with at least 1 ARFI symptom at (1) 3 years (n = 10 219), (2) 8 years (n = 4430), (3) 3 or 8 years (ie, ever; n = 13 128); and (4) both 3 and 8 years (ie, persistent; n = 1521). Cases for GWAS of ARFI-clinical included children with at least 1 symptom at (1) 3 or 8 years and clinical significance indicator (ie, ever; n = 2336) and (2) both 3 and 8 years and clinical significance indicator (ie, persistent; n = 452). Controls comprised children with no ARFI-broad at any age and no eating disorder or relevant general medical condition diagnoses in NPR (n = 26 107). The study participant flow chart and case/control phenotypes for GWAS and LDSC analyses are depicted, respectively, in eFigure 1 and eTable 2 in Supplement 1.

**Table 2.** Avoidant/Restrictive Food Intake (ARFI) Symptoms and Clinical Significance Indicators in Children With Persistent, Transient, and Emergent ARFI

Symptom	No. (%)		
	ARFI–broad persistent (n = 2129 [5.96%])	ARFI–broad transient (n = 6338 [17.73%])	ARFI–broad emergent (n = 3001[8.39%])
ARFI symptoms: age 3 y			
Does not eat well	608 (28.56)	1070 (16.88)	NA
Not happy eating food	214 (10.05)	400 (6.31)	NA
Fussy	1281 (60.17)	2402 (37.90)	NA
Careful to make sure child eats enough	572 (26.87)	1306 (20.61)	NA
If child says not hungry, try to get him/her to eat	767 (36.03)	3069 (48.42)	NA
Child needs guidance or regulation to eat enough	758 (35.60)	1597 (25.20)	NA
ARFI symptoms: age 8 y			
Does not enjoy tasting new foods	600 (28.18)	NA	422 (14.06)
Gets full easily	432 (20.29)	NA	470 (15.66)
Eats slowly	615 (28.89)	NA	813 (27.09)
Takes more than 30 min to finish meal	88 (4.13)	NA	63 (2.10)
Gets full before finished meal	311 (14.61)	NA	448 (14.93)
Does not enjoy a variety of foods	656 (30.81)	NA	461 (15.36)
Is not interested in tasting new food	811 (38.09)	NA	689 (22.96)
Eats less when upset	190 (8.92)	NA	456 (15.19)
Leaves food on plate at the end of a meal	297 (13.95)	NA	335 (11.16)
Eats less when angry	231 (10.85)	NA	482 (16.06)
Clinical significance indicators			
Any clinical indicator (ARFI-clinical)	624 (29.31)	1157 (18.25)	484 (16.13)
Type of clinical indicator			
Weight loss/failure to grow^a^	481 (22.59)	943 (14.88)	363 (12.10)
Nutritional deficiency^b^	138 (6.48)	232 (3.66)	110 (3.67)
Clinical diagnosis of eating disorder^c^	152 (7.14)	105 (1.66)	61 (2.03)

Abbreviations: *ICD-10*, *International Statistical Classification of Diseases and Related Health Problems, Tenth Revision*; *ICPC-2*, *International Classification of Primary Care, Second Revision*; NA, not applicable.

^a^ This was defined by mother-reported too little weight gain (either now or previously) for child age 3; body mass index less than 5th percentile based on mother-reported height and weight items for child ages 3 and 8 years; and diagnostic codes from *ICPC-2* (T08, T10) and *ICD-10* (R63.4, E43, E45).

^b^ This was assessed with diagnostic codes from *ICPC-2* (T91, B81) and *ICD-10* (E40, E41, E42, E44, E46, E50, E51, E53, E55, E56, E58, E59, E60, E61, E63, E64).

^c^ This was assessed with diagnoses from *ICPC-2* (T03, T04, P11) and *ICD-10* (F50.8, F50.9, F98.2, R63.0, R63.3, R63.6). All diagnoses were required to have been first assigned between 2 and 10 years of age. Of the 35 751 children included in the sample, 1.8% were classified with ARFI-clinical persistent, 3.2% with ARFI-clinical transient, and 1.4% with ARFI-clinical emergent.

Of all children with available data at 3 and 8 years, 2265 (6.3%) were further classified with ARFI-clinical: 624 (1.8%) were classified with persistent, 1157 (3.2%) with transient, and 484 (1.4%) with emergent.

### Developmental Characteristics

**Figure 1.**
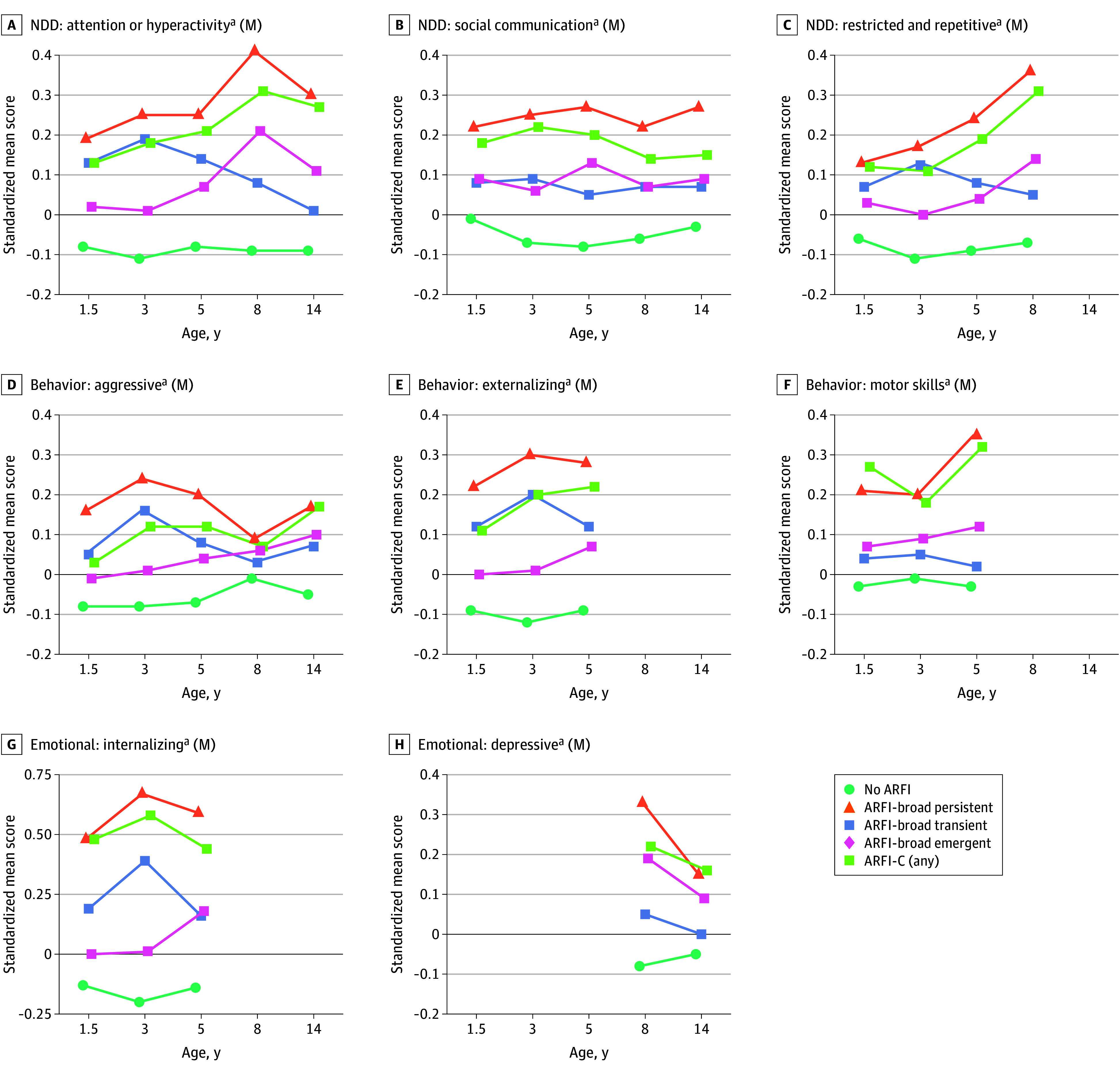
Developmental Characteristics for Children With and Without Avoidant/Restrictive Food Intake (ARFI) Phenotypes (N = 35 751) Higher scores reflect greater difficulties or higher levels of the behavior for each characteristic. Characteristics marked with (M) are mother reported. Ages for each group are as follows: ARFI–broad persistent (3 and 8 years), ARFI–broad transient (3 years), ARFI–broad emergent (8 years), and ARFI-clinical (any subtype). Figure 1 presents a subset of all developmental characteristics; the full set is reported in eTable 5 in Supplement 1. NDD indicates neurodevelopmental difficulties. ^a^Statistically significant differences were identified using independent samples *t* tests (α = .05), with *P* values adjusted for the false discovery rate. Each scale was standardized (ie, with mean = 0, SD = 1) based on all available data in the Norwegian Mother, Father, and Child Cohort Study.

Children classified with ARFI–broad persistent exhibited a higher level of mother-reported difficulties in eating, language development, motor skills, emotional problems, attention/hyperactivity, and restricted/repetitive and aggressive/uncooperative behaviors from infancy through age 14 years (Figure 1 and eTable 5 in Supplement 1). Group differences (comparing children with ARFI–broad persistent and no ARFI-broad) were statistically significant at all ages for mother-reported measures (eTable 6 in Supplement 1). At 14 years, children with ARFI–broad persistent reported less prosocial behavior. Maternal sociodemographic characteristics are reported in eTable 7 in Supplement 1.

Descriptive trends indicated that children classified with ARFI–broad transient and emergent generally exhibited intermediate levels of difficulties compared with those with ARFI–broad persistent and no ARFI-broad (Figure 1 and eTable 5 in Supplement 1). The highest level of difficulties was observed for ARFI–clinical persistent (eTable 8 in Supplement 1).

Children with ARFI–broad persistent had a higher prevalence of intellectual disability, global developmental delay, autism, attention-deficit/hyperactivity disorder (ADHD), and epilepsy compared with children with no ARFI-broad (Table 3). Diagnosis prevalence for ARFI-clinical groups is reported in eTable 9 in Supplement 1.

**Table 3.** Lifetime Diagnoses Among Children With and Without Avoidant/Restrictive Food Intake (ARFI) Phenotypes (N = 35 751)

Diagnoses	No. (%)				
	No ARFI-broad (n = 24 283)	ARFI–broad persistent (n = 2129)	ARFI–broad transient (n = 6338)	ARFI–broad emergent (n = 3001)	Any ARFI-clinical (n = 2265)
Intellectual disability (F70-F79)^a^	88 (0.36)	32 (1.50)	36 (0.57)	24 (0.80)	47 (2.08)
Global developmental delay (F83)^a^	91 (0.37)	21 (0.99)	33 (0.52)	24 (0.80)	31 (1.37)
OCD (F42)	260 (1.07)	29 (1.36)	62 (0.98)	51 (1.70)	44 (1.94)
Autism (F84.0, F84.1, F84.5, F84.8, F84.9)^a^	412 (1.70)	117 (5.50)	133 (2.10)	108 (3.60)	122 (5.39)
ADHD (F90)^a^	1292 (5.32)	202 (9.49)	400 (6.31)	271 (9.03)	231 (10.20)
Anorexia nervosa (F50.0 and F50.1)	301 (1.24)	27 (1.27)	54 (0.85)	44 (1.47)	25 (1.10)
Other eating disorders (F50.2, F50.3, F50.4, F50.5)	56 (0.23)	<5	10 (0.16)	5 (0.17)	<5
Neurological: Epilepsy (G40)^a^	324 (1.33)	48 (2.25)	102 (1.61)	58 (1.93)	73 (3.22)
Gastrointestinal: Crohn’s disease (K50), ulcerative colitis (K51), irritable bowel syndrome (K58), celiac disease (K90.0)	933 (3.84)	99 (4.65)	242 (3.82)	138 (4.60)	154 (6.80)

Abbreviations: ADHD, attention-deficit/hyperactivity disorder; *ICD-10*, *International Statistical Classification of Diseases and Related Health Problems*,* Tenth Revision*; OCD, obsessive-compulsive disorder.

^a^ Diagnoses for which 2-proportion *z* tests yielded statistically significant differences in prevalence (α = .05) with *P* values corrected for the false discovery rate (9 tests), when comparing children with ARFI–broad persistent and no ARFI-broad. All diagnostic codes in Table 3 are reported according to *ICD-10*.

### Genetic Associations With ARFI

#### SNV Heritability

SNV heritability (SNV-*h*^2^) estimates on the liability scale ranged from 8% to 16% and were statistically significant for ARFI-broad at 3 years (SNV-*h*^2^ = 0.08; SE = 0.03; *P* = .001), 8 years (SNV-*h*^2^ = 0.12; SE = 0.04; *P* = .003), and 3 or 8 years (SNV-*h*^2^ = 0.08; SE = 0.02; *P* <.001), and ARFI-clinical at 3 or 8 years (SNV-*h*^2^ = 0.16; SE = 0.06; *P* = .004). Sensitivity analyses with more stringent inclusion criteria also yielded statistically significant SNV-*h*^2^ estimates for these phenotypes (eTable 10 in Supplement 1).

#### GWAS and Gene-Based Association Analyses

**Figure 2.**
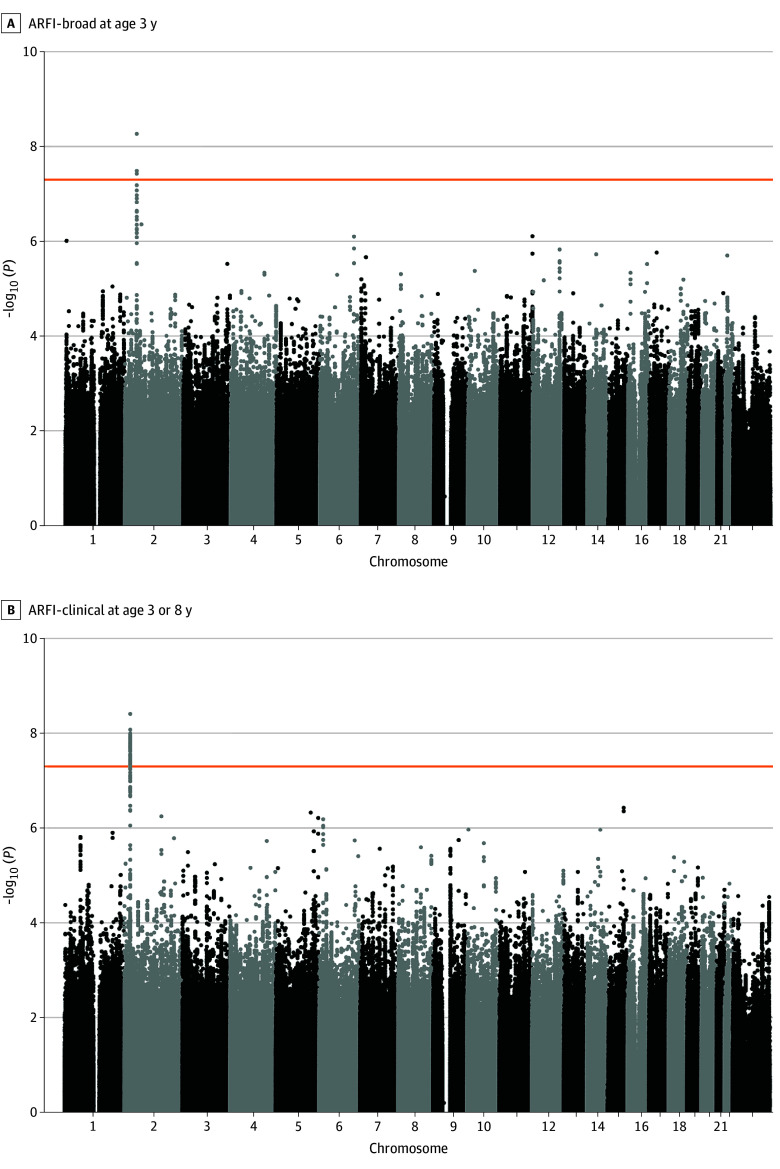
Manhattan Plots A, Avoidant/restrictive food intake (ARFI)–broad at 3 years. B, ARFI-clinical at 3 or 8 years. Manhattan plots of the genome-wide association study of ARFI phenotypes. The y-axis indicates the −log10 *P* value for each single single-nucleotide variant (chromosomal position on the x-axis). The orange line indicates genome-wide significance (*P* < 5 × 10^−8^). For ARFI-broad 3 years, the number of cases and controls were 10 219 and 26 107, respectively. For ARFI-clinical 3 or 8 years, the number of cases and controls were 2336 and 26 107, respectively.

GWAS and conditional analyses identified 1 independent genome-wide significant locus for ARFI-broad at age 3 years (Figure 2), located at rs6545025 on chromosome 2 (base pair position: 48 492 218; B = −0.12; SE = 0.02; *P* = 5.51 × 10^−9^). Additionally, 1 independent significant locus was identified for ARFI-clinical at 3 or 8 years, mapped to rs11676272 on chromosome 2 (base pair position: 25 141 538; B = −0.18; SE = 0.03; *P* = 3.98 × 10^−9^). Manhattan plots for all phenotypes and zoom locus plots for the 2 genome-wide significant loci are included in (eFigures 2-13 in Supplement 1).

One gene, *ADCY3*, located on chromosome 2, was associated with ARFI-clinical at 3 or 8 years (*z* = 5.42; *P* = 3.03 × 10^−8^) after correcting for multiple testing (eTable 11 in Supplement 1). This gene included 49 SNVs in the analysis and exceeded the Bonferroni-adjusted threshold for genome-wide significance. No other genes reached statistical significance in this analysis.

#### Genetic Correlations

For both ARFI-broad (3 years, 8 years, 3 or 8 years) and ARFI-clinical (3 or 8 years), we identified small to moderate genetic correlations with educational attainment (−0.15 to −0.37), body mass index (BMI) (−0.17 to −0.31), and food-predilection phenotypes (acquired, low caloric, savory; −0.22 to −0.43), and large for childhood BMI (−0.55 to −0.94) (eTables 12 and 13 in Supplement 1). For both ARFI-broad (3 years, 3 or 8 years) and ARFI-clinical (3 or 8 years), we observed a negative genetic correlation with cognitive ability (−0.21 to −0.29). There was a moderate genetic correlation between ARFI-broad (8 years) and inflammatory bowel disease (0.19), ARFI-broad (3 years, 8 years, 3 or 8 years) and ulcerative colitis (0.23-0.26), and ARFI-clinical (3 or 8 years) and celiac disease (0.49). Additionally, there was a moderate genetic correlation between ARFI-clinical (3 or 8 years) and ADHD (0.21), and both ARFI-broad (3 years, 8 years, 3 or 8 years) and ARFI-clinical (3 or 8 years) and the food-predilection phenotype caffeinated sweet drinks (0.36-0.51). The genetic correlation between ARFI-broad at 3 and 8 years was 0.53.

## Discussion

We used data from a large pregnancy-based cohort study^25^ to estimate the prevalence of ARFI in children aged 3 and 8 years, examine developmental characteristics from early childhood through adolescence, and investigate genetic associations of ARFI phenotypes using genome-wide methods.

Prevalence estimates ranged from 6% for ARFI–broad persistent (present at both 3 and 8 years) to 18% for ARFI–broad transient (3 years only). These figures align with prevalence estimates for picky eating, such as 10% to 15% reported in the Avon Longitudinal Study of Parents and Children (ALSPAC),^2^ and 6% for persistent picky eating in the Generation R study.^3^ The proportion of children with ARFI and clinical significance indicators was 1.8% for ARFI–clinical persistent, 3.2% for ARFI–clinical transient, and 1.4% for ARFI–clinical emergent (8 years only), in agreement with recent ARFID (diagnosis and symptoms) prevalence estimates (range, 2%-6%).^12,21^ Our findings also highlight heterogeneity in ARFI symptom patterns: although most affected children exhibited symptoms only at ages 3 or 8 years, 19% of children with symptoms were classified with a persistent pattern (ie, present at both ages).

Children with ARFI–broad persistent exhibited more difficulties across multiple developmental domains, consistent with studies reporting increased difficulties in related developmental areas, including emotional, attentional, and social challenges, among children with ARFID^11,12,39^ and persistent picky eating.^3^ This highlights the need for early identification and targeted support across multiple developmental domains for many children affected by ARFI. The heightened developmental difficulties observed for children with ARFI–broad persistent, also when compared descriptively with ARFI–clinical transient and emergent, may indicate that a more chronic course of avoidant/restrictive food intake is associated with greater cumulative impact on developmental domains.

Children with ARFI–broad persistent had more than double the prevalence of lifetime autism diagnoses and nearly double the prevalence of ADHD diagnoses compared with children who never experienced this eating pattern. This aligns with well-established evidence of high co-occurrence between ARFID and neurodevelopmental conditions, including ADHD and autism diagnoses and traits.^12,13,40,41,42^

We also extend previous research identifying a strong genetic component to eating- and feeding-associated phenotypes in childhood^16,17,18^ and ARFID.^21^ SNV-*h*^2^ ranged from 8% to 16%. Estimates were highest for ARFI with clinical indicators (ie, ARFI-clinical), which may correspond most closely with ARFID. For comparison, this is higher than reported SNV-*h*^2^ for internalizing symptoms in childhood and adolescence (5.6%)^43^ and similar to SNV-*h*^2^ for parent-reported ADHD symptoms (range, 5%-14%).^44^

Furthermore, we identified a significant association between ARFI-clinical and the missense variant rs11676272 at *ADCY3*, which has been previously implicated in obesity across different populations,^45,46^ olfactory signaling,^47^ and inflammatory bowel disease.^48^ The lead variant shows strong association with BMI already from infancy and throughout childhood.^49^ Future studies are needed to replicate associations between individual genetic variants and avoidant/restrictive eating and investigate the biological mechanisms underlying these associations.

Finally, we observed genetic correlations between both ARFI-broad and ARFI-clinical and several phenotypes. Thus, ARFI phenotypes are partly influenced by genetic variants that are also associated with other complex traits, including mental health, cognition/education, anthropometric and food-associated characteristics, and gastrointestinal function. This aligns with well-established evidence of genetic correlations across many complex traits.^50^ Future studies are needed to understand which mechanisms underlie these genetic correlations, including potential pleiotropy, unidirectional causal effects or bidirectional causal effects.

### Strengths and Limitations

Our study has several strengths, including the use of a large population-based pregnancy cohort to assess mother-reported ARFI and developmental characteristics across childhood, in combination with health registry data to identify plausible clinical indicators. We conducted comprehensive genome-wide analyses, offering new insights into the genetic underpinnings of avoidant and restrictive food intake symptoms.

Our study has important limitations. First, we focused on avoidant and restrictive food intake, which is a core symptom of ARFID but does not capture all diagnostic criteria required for ARFID. Specifically, the measures administered in the MoBa study did not allow us to assess concerns about aversive consequences of eating, interference with psychosocial functioning, or if the eating disturbance(s) was explained by lack of food, culturally sanctioned practices, or body weight or shape concerns. Therefore, phenotypes should not be interpreted as ARFID diagnoses, but as ARFID traits, accompanied by additional co-occurring indicators of clinical significance for ARFI-clinical. Second, the response rate in the MoBa study was 41%, and there is substantial attrition, which could introduce selection or attrition biases.^51,52^ Less data available for children aged 8 years could have reduced the statistical power of the genetic analyses at this age. Third, the genomic pipeline in the MoBa study is currently limited to participants of European ancestry,^26^ limiting the generalizability of findings from the genetic analyses. Future studies should examine the genetic underpinnings of avoidant/restrictive eating in more diverse samples. Fourth, the variance explained by common SNVs was modest (SNV-*h*^2^ ranged from 8%-16%), which indicates that the statistical power to detect associations with individual SNVs with very small effect sizes may be limited. Furthermore, few participants included in the ARFI-broad and ARFI-clinical persistent groups limits the statistical power to detect associations with genetic variants.

## Conclusions

In a large general pediatric population sample, the prevalence of different ARFI phenotypes ranged from 6% to 18%, narrowing to 2% to 3% when limiting to children with clinical significance indicators. Children with persistent ARFI exhibited more parent-reported difficulties across multiple developmental domains from infancy to adolescence. SNV-*h*^2^ estimates ranged from 8% to 16%, and an association was identified with *ADCY3* for ARFI with clinical indicators. Our findings underscore the importance of early identification and support for children affected by persistent ARFI. The discovery of 2 genome-wide significant loci, along with genetic overlap with psychological, cognitive and educational, anthropometric, food-associated, and gastrointestinal traits, represents an important step toward characterizing the genetic architecture of avoidant and restrictive food intake in childhood.

## Notes

Supplement 1.
eMethods.
**eFigure 1.** Flow Chart for Identifying ARFI-Broad and ARFI-Clinical Phenotypes **eTable 1.** Items Used to Develop the Avoidant Restrictive Food Intake Phenotypes (ARFI-Broad and ARFI-Clinical) **eTable 2.** Case and Control Phenotypes for GWAS and LDSC Analyses **eTable 3.** Sources of Relevant GWAS Summary Statistics **eTable 4.** Preregistration Deviations Table **eTable 5.** Developmental Characteristics for Children With and Without Avoidant/Restrictive Food Intake (ARFI) Phenotypes **eTable 6.** Test Statistics Comparing Children With ARFI-Persistent and No ARFI-Broad **eTable 7.** Maternal Sociodemographic Characteristics at MoBa Q1 for Children With No ARFI-Broad and Children With ARFI-Broad Persistent **eTable 8.** Characteristics Among Children With ARFI-Clinical **eTable 9.** Lifetime Diagnoses Among Children With ARFI-Clinical **eTable 10.** SNV-Heritability Estimates, Sensitivity Analyses With More Stringent Inclusion Criteria **eTable 11.** Results From Gene-Based Association Analyses **eTable 12.** Genetic Correlation Estimates **eTable 13.** Genetic Correlation Estimates, Sensitivity Analyses With More Stringent Inclusion Criteria **eFigure 2.** Manhattan Plot: ARFI-Broad Emergent (8 Years) **eFigure 3.** Manhattan Plot: ARFI-Broad at 3 or 8 Years **eFigure 4.** Manhattan Plot: ARFI-Broad Persistent **eFigure 5.** Manhattan Plot: ARFI-Clinical Persistent **eFigure 6.** Manhattan Plot: ARFI-Broad Transient (3 Years), Sensitivity Analysis **eFigure 7.** Manhattan Plot: ARFI-Broad Emergent (8 Years), Sensitivity Analysis **eFigure 8.** Manhattan Plot: ARFI-Broad at 3 or 8 Years, Sensitivity Analysis **eFigure 9.** Manhattan Plot: ARFI-Broad Persistent, Sensitivity Analysis **eFigure 10.** Manhattan Plot: ARFI-Clinical at 3 or 8 Years, Sensitivity Analysis **eFigure 11.** Manhattan Plot: ARFI-Clinical Persistent, Sensitivity Analysis **eFigure 12.** Regional Plot: ARFI-Broad Transient (3 Years) **eFigure 13.** Regional Plot: ARFI-Clinical at 3 or 8 Years
eReferences.

Supplement 2.
Data Sharing Statement.
