# Supplementary material for: Prevalence, Characteristics, and Genetic Architecture of Avoidant/Restrictive Food Intake Phenotypes
Source: JAMA Pediatr. 2025 Nov 24;180(1):45–55. doi: 10.1001/jamapediatrics.2025.4786 (PMC12645403; doi:10.1001/jamapediatrics.2025.4786)
Supplement: Supplement 1. — eMethods. eFigure 1. Flow Chart for Identifying ARFI-Broad and ARFI-Clinical Phenotypes eTable 1. Items Used to Develop the Avoidant Restrictive Food Intake Phenotypes (ARFI-Broad and ARFI-Clinical) eTable 2. Case and Control Phenotypes for GWAS and LDSC Analyses eTable 3. Sources of Relevant GWAS Summary Statistics eTable 4. Preregistration Deviations Table eTable 5. Developmental Characteristics for Children With and Without Avoidant/Restrictive Food Intake (ARFI) Phenotypes eTable 6. Test Statistics Comparing Children With ARFI-Persistent and No ARFI-Broad eTable 7. Maternal Sociodemographic Characteristics at MoBa Q1 for Children With No ARFI-Broad and Children With ARFI-Broad Persistent eTable 8. Characteristics Among Children With ARFI-Clinical eTable 9. Lifetime Diagnoses Among Children With ARFI-Clinical eTable 10. SNV-Heritability Estimates, Sensitivity Analyses With More Stringent Inclusion Criteria eTable 11. Results From Gene-Based Association Analyses eTable 12. Genetic Correlation Estimates eTable 13. Genetic Correlation Estimates, Sensitivity Analyses With More Stringent Inclusion Criteria eFigure 2. Manhattan Plot: ARFI-Broad Emergent (8 Years) eFigure 3. Manhattan Plot: ARFI-Broad at 3 or 8 Years eFigure 4. Manhattan Plot: ARFI-Broad Persistent eFigure 5. Manhattan Plot: ARFI-Clinical Persistent eFigure 6. Manhattan Plot: ARFI-Broad Transient (3 Years), Sensitivity Analysis eFigure 7. Manhattan Plot: ARFI-Broad Emergent (8 Years), Sensitivity Analysis eFigure 8. Manhattan Plot: ARFI-Broad at 3 or 8 Years, Sensitivity Analysis eFigure 9. Manhattan Plot: ARFI-Broad Persistent, Sensitivity Analysis eFigure 10. Manhattan Plot: ARFI-Clinical at 3 or 8 Years, Sensitivity Analysis eFigure 11. Manhattan Plot: ARFI-Clinical Persistent, Sensitivity Analysis eFigure 12. Regional Plot: ARFI-Broad Transient (3 Years) eFigure 13. Regional Plot: ARFI-Clinical at 3 or 8 Years eReferences. [file jamapediatr-e254786-s001.pdf]

Supplementary Online Content

Bjørndal LD, Corfield EC, Hannigan LJ, et al. Prevalence, characteristics, and genetic architecture of avoidant restrictive food intake phenotypes. *JAMA Pediatr*. Published online November 24, 2025. doi:10.1001/jamapediatrics.2025.4786

eMethods.

eFigure 1. Flow Chart for Identifying ARFI-Broad and ARFI-Clinical Phenotypes

eTable 1. Items Used to Develop the Avoidant Restrictive Food Intake Phenotypes (ARFI-Broad and ARFI-Clinical)

eTable 2. Case and Control Phenotypes for GWAS and LDSC Analyses

eTable 3. Sources of Relevant GWAS Summary Statistics

eTable 4. Preregistration Deviations Table

eTable 5. Developmental Characteristics for Children With and Without Avoidant/Restrictive Food Intake (ARFI) Phenotypes

eTable 6. Test Statistics Comparing Children With ARFI-Persistent and No ARFI-Broad

eTable 7. Maternal Sociodemographic Characteristics at MoBa Q1 for Children With No ARFI-Broad and Children With ARFI-Broad Persistent

eTable 8. Characteristics Among Children With ARFI-Clinical

eTable 9. Lifetime Diagnoses Among Children With ARFI-Clinical

eTable 10. SNV-Heritability Estimates, Sensitivity Analyses With More Stringent Inclusion Criteria

eTable 11. Results From Gene-Based Association Analyses

eTable 12. Genetic Correlation Estimates

eTable 13. Genetic Correlation Estimates, Sensitivity Analyses With More Stringent Inclusion Criteria

eFigure 2. Manhattan Plot: ARFI-Broad Emergent (8 Years)

eFigure 3. Manhattan Plot: ARFI-Broad at 3 or 8 Years

eFigure 4. Manhattan Plot: ARFI-Broad Persistent

eFigure 5. Manhattan Plot: ARFI-Clinical Persistent

eFigure 6. Manhattan Plot: ARFI-Broad Transient (3 Years), Sensitivity Analysis

eFigure 7. Manhattan Plot: ARFI-Broad Emergent (8 Years), Sensitivity Analysis

© 2025 Bjørndal LD et al. *JAMA Pediatrics*.

eFigure 8. Manhattan Plot: ARFI-Broad at 3 or 8 Years, Sensitivity Analysis

eFigure 9. Manhattan Plot: ARFI-Broad Persistent, Sensitivity Analysis

eFigure 10. Manhattan Plot: ARFI-Clinical at 3 or 8 Years, Sensitivity Analysis

eFigure 11. Manhattan Plot: ARFI-Clinical Persistent, Sensitivity Analysis

eFigure 12. Regional Plot: ARFI-Broad Transient (3 Years)

eFigure 13. Regional Plot: ARFI-Clinical at 3 or 8 Years

eReferences.

This supplementary material has been provided by the authors to give readers additional information about their work.

© 2025 Bjørndal LD et al. *JAMA Pediatrics*.

eMethods.

Measures Used to Assess Developmental Characteristics

Developmental characteristics were assessed using multiple measures administered in MoBa[1] across 14 years of follow-up: Eating-related difficulties with the Child Behavior Checklist (CBCL), Child Feeding Questionnaire (CFQ), Child Eating Behavior Questionnaire (CEBQ), and Eating Disorder Examination-Questionnaire (EDE-Q); language development using the Ages and Stages Questionnaire (ASQ) and MoBa items; motor skills with the ASQ, Child Development Inventory (CDI), and MoBa items; social communication and prosocial behavior using the Modified Checklist for Autism in Toddlers (M-CHAT), Social Communication Questionnaire (SCQ), Childhood Autism Spectrum Test (CAST), and Strengths and Difficulties Questionnaire (SDQ); restricted and repetitive behaviors using M-CHAT, SCQ, and CAST; emotional and behavioral difficulties with CBCL, Short Mood and Feelings Questionnaire (SMFQ), Screen for Child Anxiety Related Emotional Disorders (SCARED), Parent/Teacher Rating Scale for Disruptive Behavior Disorders (RS-DBD), and Hopkins Symptom Checklist (SCL-10); and attention/hyperactivity difficulties with CBCL and RS-DBD.

© 2025 Bjørndal LD et al. *JAMA Pediatrics*.

eFigure 1.

Identification of ARFI-Broad and ARFI-Clinical for Phenotypic and Genetic Analyses.

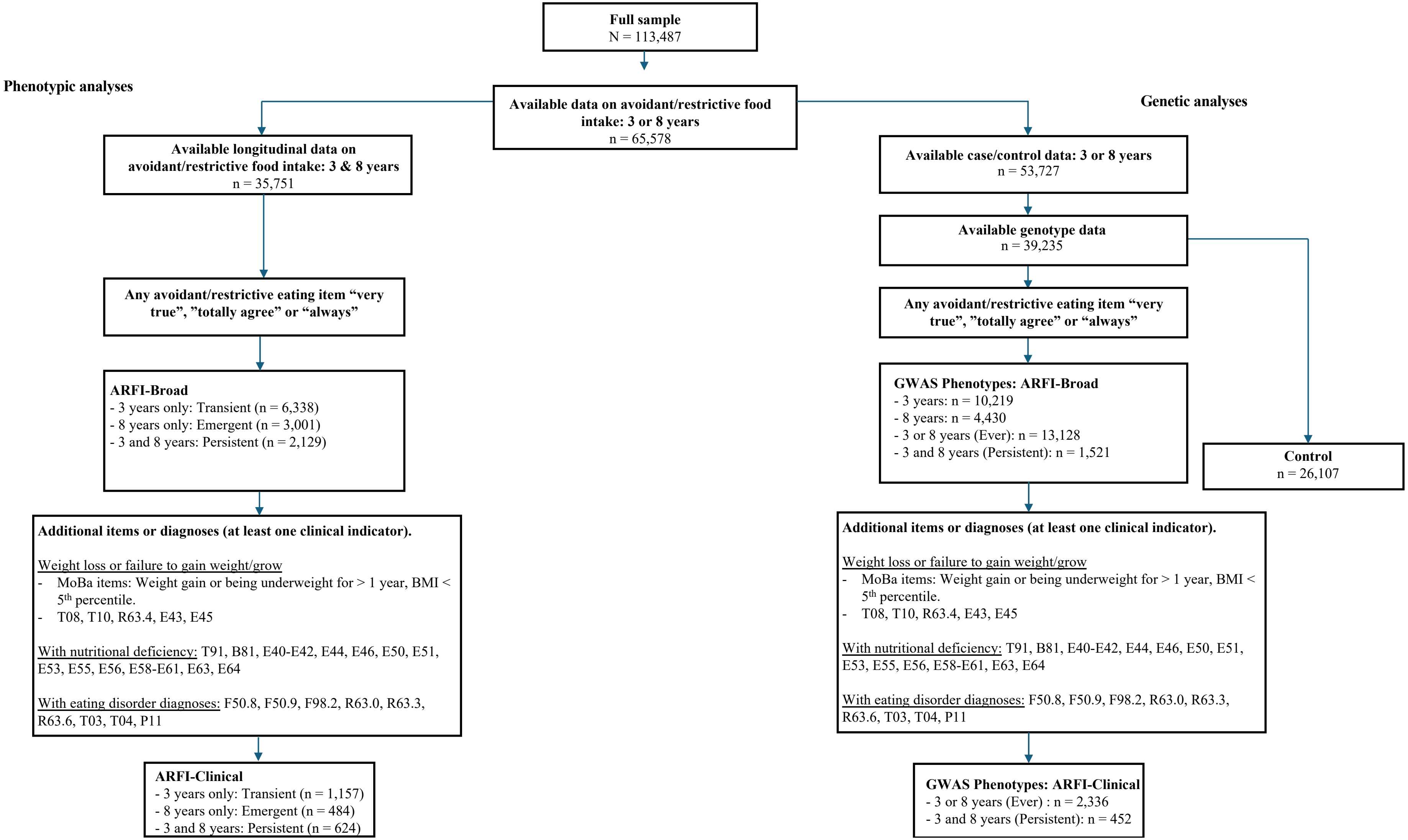

© 2025 Bjørndal LD et al. *JAMA Pediatrics*.

eTable 1.

Items Used to Develop the Avoidant Restrictive Food Intake Phenotypes (ARFI-Broad and ARFI-Clinical).

Age	Items	Response category	Scale
3 years	1) Doesn't eat well	Very true	CBCL[a]
	2) Is very fussy when it comes to food	Very true	ITSEA[b]
	3) I have to be especially careful to make sure that my child eats enough	Very true	CFQ[c]
	4) If my child says: "I'm not hungry", I try to get him/her to eat anyway	Totally agree	CFQ[c]
	5) If I did not guide or regulate my child's eating, he/she would eat much less than he/she should	Totally agree	CFQ[c]
	6) Doesn't seem to be happy eating food (don't include sweets)	Totally agree	CBCL[a]
8 years	1) My child enjoys tasting new foods (R)	Never	CEBQ[d]
	2) My child gets full easily	Always	CEBQ[d]
	3) My child eats slowly	Always	CEBQ[d]
	4) My child takes more than 30 minutes to finish a meal	Always	CEBQ[d]
	5) My child gets full before his/her meal is finished	Always	CEBQ[d]
	6) My child enjoys a wide variety of foods (R)	Never	CEBQ[d]
	7) My child is interested in tasting food s/he hasn't tasted before (R)	Never	CEBQ[d]
	8) My child eats less when upset	Always	CEBQ[d]
	9) My child leaves food on his/her plate at the end of a meal	Always	CEBQ[d]
	10) My child eats less when angry	Always	CEBQ[d]

Note. [a]Child Behaviour Checklist; [b]The Infant-Toddler Social and Emotional Assessment; [c]Child Feeding Questionnaire; [d]The Children's Eating Behaviour Questionnaire. R: Reversed.

© 2025 Bjørndal LD et al. *JAMA Pediatrics*.

eTable 2.

Case and Control Phenotypes for GWAS and LDSC Analyses.

Cases[a]	Description
ARFI-Broad: 3 years (n = 10,219)	At least one symptom at 3 years
ARFI-Broad: 8 years (n = 4,430)	At least one symptom at 8 years
ARFI-Broad: 3 or 8 years (n = 13,128)	At least one symptom at 3 or 8 years
ARFI-Broad: Persistent (n = 1,521)	At least one symptom at 3 and 8 years
ARFI-Clinical: 3 or 8 years (n = 2,336)	At least one symptom (3 or 8 years) and clinical indicator(s)
ARFI-Clinical: Persistent (n = 452)	At least one symptom (3 and 8 years) and clinical indicator(s)
**Control**	**Description**
Control: Broad (n = 26,107)	No symptoms reported at 3 or 8 years; no clinical indicators at 3 or 8 years; no anorexia nervosa, other eating disorder[b], or general medical[a] diagnoses.

Note. [a]In sensitivity analyses, we additionally excluded children with the following general medical diagnoses which could plausibly explain the dysregulated eating pattern (ICD-10) as cases: C18-C21, C33, C34, C38, C45, C50, C53, C61, C77-C85, C88, C90, C96, D01, D02, D05-D07, E03, E10, E14, E78, G80-G83, K50, K51, K56, K59, K63, K90. [b]ICD-10: F50.2, F50.3, F50.4, F50.5, F50.8, F50.9.

© 2025 Bjørndal LD et al. *JAMA Pediatrics*.

eTable 3.

Sources of Relevant GWAS Summary Statistics.

Phenotype	GWAS
OCD	International Obsessive Compulsive Disorder Foundation Genetics Collaborative (IOCDF-GC) & OCD Collaborative Genetics Association Studies (OCGAS)[2]
Autism	Grove et al.[3]
ADHD	Demontis et al.[4]
AN	Watson et al.[5]
Anxiety	Otowa et al.[6]
Epilepsy (all)<br>Epilepsy (eu)	International League Against Epilepsy Consortium on Complex Epilepsies[7]
Cognitive ability	Savage et al.[8]
Educational attainment	Lee et al.[9]
Birth weight	Warrington et al.[10]
BMI	Yengo et al.[11]
Childhood BMI	Vogelezang et al.[12]
Childhood BMI 2025	Downie et al.[13]
IBS	Eijsbouts et al.[14]
IBD	de Lange et al.[15]
Ulcerative colitis	de Lange et al.[15]
Crohn's disease	de Lange et al.[15]
Celiac disease	Trynka et al.[16]
Food-liking: Acquired	May-Wilson et al.[17]
Food-liking: Caffeinated sweet drinks	May-Wilson et al.[17]
Food-liking: Highly palatable	May-Wilson et al.[17]
Food-liking: Low caloric	May-Wilson et al.[17]
Food-liking: Savoury	May-Wilson et al.[17]
Leptin	Kilpeläinen et al.[18]
Leptin adj. BMI	Kilpeläinen et al.[18]

Note. OCD: Obsessive-compulsive disorder. ADHD: Attention-deficit/hyperactivity disorder. AN: Anorexia nervosa. BMI: Body Mass Index. IBS: Irritable bowel syndrome. IBD: Inflammatory Bowel Disease.

Overview of deviations from preregistration

We deviated from the pre-registration (available on the Open Science Framework: https://osf.io/m5hvs). We provide an overview of these deviations and corresponding justifications in Table S4, using a template from Willroth & Atherton.[19]

© 2025 Bjørndal LD et al. *JAMA Pediatrics*.

eTable 4.

Preregistration Deviations Table.

**Deviations**					
#	Details		Original Wording	Deviation Description	Reader Impact
1	Type	Analysis	We originally aimed to conduct an exploratory factor analysis to determine which items adequately captured avoidant and restrictive food intake (ARFI) and use a sum score approach to classifying children with ARFI.	We deviated from the preregistered approach to use an item-based approach, to more closely align with current diagnostic systems and practices for ARFID. At 3 years, if parents reported either 'very true' (for items 1-3) or 'totally agree' (for items 4-6) for at least one item, children were classified with ARFI-Broad at this age. At 8 years, if parents reported (for at least one of the items) 'never' (for items 1, 6 and 7) or 'always' (items 2-5 and 8-10), children were classified with ARFI-Broad at this age. The change of this classification approach was made following discussions with collaborators after pre-registration.<br><br>As we changed the classification approach to binary, we did not perform correlational analyses to examine stability in ARFI, which were pre-registered based on the original sum score approach.	This deviation should not affect the readers' interpretation of the study and/or results.
	Reason	New knowledge			
	Timing	After data access			

© 2025 Bjørndal LD et al. *JAMA Pediatrics*.

2	Type	Analysis	We originally planned to include two separate clinical indicators for eating disorder (ED) diagnoses which required parent-reported avoidant/restrictive eating (clinical indicator C in the preregistration) and diagnoses which did not require parent-reported avoidant/restrictive eating (clinical indicator D in the preregistration).	These indicators were combined for the final manuscript to reduce the number of clinical indicators and the length of the manuscript.	This deviation should not affect the readers' interpretation of the study and/or results.
	Reason	New knowledge			
	Timing	After data access			
3	Type	Analysis	The ICPC-2 diagnostic code T04 (child eating difficulties) was included in the preregistration for classifying children with both the clinical indicators 'nutritional deficiency' and 'other eating disorder diagnoses'.	To avoid overlap with children being possibly assigned multiple clinical indicators based on this one diagnostic code, we only included this diagnostic code for the clinical indicator 'other eating disorder diagnoses' for the analyses reported in the manuscript.	This deviation should not affect the readers' interpretation of the study and/or results.
	Reason	Typo/error			
	Timing	After data access			
4	Type	Analysis	Several diagnoses to be used for classification with the clinical significance indicator 'nutritional deficiency' were not available in the final data used for analyses (ICD-10): E52, E54, E57.	These were omitted from all analyses.	This deviation should not affect the readers' interpretation of the study and/or results. We note that the inclusion of these diagnoses (if available) could have contributed to an increase in the number identified with the indicator.
	Reason	Plan not possible			
	Timing	After data access			
5	Type	Analysis	Several diagnoses which were to be used in sensitivity analyses were not available in the final data used for analyses (listed in Table 5 in the pre-registration).	These were omitted from all analyses.	This deviation should not affect the readers' interpretation of the study and/or results. We note that the inclusion of these diagnoses could have contributed to an increase in the number excluded in the sensitivity analyses.
	Reason	Plan not possible			
	Timing	After data access			

© 2025 Bjørndal LD et al. *JAMA Pediatrics*.

6	Type	Analysis	In the pre-registration, we aimed to report the prevalence of global developmental delay using other indicators in addition to F83 (R62, MoBa items).	The other pre-registered indicators were not suitable to indicate global developmental delay.	This deviation should not affect the readers' interpretation of the study and/or results.
	Reason	Miscommunication			
	Timing	After data access			
7	Type	Analysis	In the pre-registration, we aimed to report cognitive and educational phenotypes.	It was not clear in the pre-registration what this referred to maternal education level and suitable indicators for cognitive phenotypes were not available.	This deviation should not affect the readers' interpretation of the study and/or results.
	Reason	Miscommunication			
	Timing	After data access			
8	Type	Analysis	In the pre-registration, we aimed to report growth phenotypes.	We did not do this to reduce the scope and length of the manuscript, and since these overlap with clinical indicators used to classify children with ARFI-Clinical.	This deviation should not affect the readers' interpretation of the study and/or results.
	Reason	New knowledge			
	Timing	After data access			
9	Type	Analysis	We pre-registered to examine genetic correlations between avoidant/restrictive eating and several phenotypes, some of which we were unable to assess, due to lack of access to GWAS summary statistics and/or limited time for analyses. This applied to the following phenotypes from the pre-registration: childhood body size, sensory perception of taste, sweet	These analyses were not conducted.	This deviation should not affect the readers' interpretation of the study and/or results.
	Reason	Plan not possible			
	Timing	After data access			

© 2025 Bjørndal LD et al. *JAMA Pediatrics*.

			taste and bitter taste, sensory and olfactory sensitivity, food addiction.		
	Type	Analysis	We incorrectly pre-registered to quantify genetic correlations with irritable bowel syndrome (IBS) only: this should also have included inflammatory bowel disease (IBD).	Analyses were conducted for IBS and IBD.	This deviation should not affect the readers' interpretation of the study and/or results.
	Reason	Typo/Error			
	Timing	After data access			
10	Type	Analysis	We originally planned to report social communication differences and restricted and repetitive behaviors using both M-CHAT and ESAT at 18 months. In the final article, we only include descriptive results from M-CHAT, as reporting findings from both measures was deemed redundant, and M-CHAT is widely used and validated.	ESAT measure not reported.	This deviation should not affect the readers' interpretation of the study and/or results.
	Reason	New knowledge			
	Timing	After data access			

Unregistered Steps

#	Details		Original Wording	Unregistered Step Description	Reader Impact
1	Type	Analysis	We also report maternal sociodemographic characteristics for ARFI-Broad Persistent and No ARFI-Broad, which was not a pre-registered descriptive analysis.	We report these characteristics using descriptive statistics.	This provides contextual information and should not affect the readers' interpretation of the study and/or results.
	Timing	After data access			
	Timing	After data access			

© 2025 Bjørndal LD et al. *JAMA Pediatrics*.

eTable 5.

Developmental Characteristics for Children With and Without Avoidant/Restrictive Food Intake (ARFI) Phenotypes (N=35,751).

Characteristics	No ARFI-broad	Persistent	Transient	Emergent	Any ARFI-Clinical
	(n = 24,283)	(n = 2,129)	(n = 6,338)	(n = 3,001)	(n = 2,265)
Male sex (registry), n (%)*	12,217 (50.43)	1,159 (54.52)	3,350 (52.92)	1,493 (49.77)	1,161 (51.26)
0.5 years					
[a]Breastfeeding problems, n (%)*	1,317 (5.43)	169 (7.95)	433 (6.84)	179 (5.97)	145 (6.40)
1.5 years					
[b]Eating difficulties*	-0.18 (0.85)	0.82 (1.28)	0.31 (1.14)	0.02 (0.98)	0.69 (1.26)
[c]Language*	-0.01 (0.99)	0.16 (1.10)	0.06 (1.03)	0.06 (1.05)	0.18 (1.12)
[c]Motor skills*	-0.03 (0.94)	0.21 (1.27)	0.04 (1.01)	0.07 (1.09)	0.27 (1.40)
[d]Social communication*	-0.01 (0.93)	0.22 (1.28)	0.08 (1.06)	0.09 (1.05)	0.18 (1.29)
[d]Restricted and repetitive*	-0.06 (0.89)	0.13 (1.04)	0.07 (1.01)	0.03 (0.95)	0.12 (1.04)
[b]Externalizing*	-0.09 (0.95)	0.22 (1.06)	0.12 (1.01)	0.00 (1.01)	0.11 (1.03)
[b]Internalizing*	-0.13 (0.90)	0.48 (1.22)	0.19 (1.06)	0.00 (1.01)	0.48 (1.20)
[b]Attention/hyperactivity*	-0.08 (0.96)	0.19 (1.06)	0.13 (1.04)	0.02 (1.02)	0.13 (1.06)
[b]Aggressive/uncooperative	-0.08 (0.96)	0.16 (1.06)	0.05 (0.99)	-0.01 (1.00)	0.03 (1.02)
3 years					
[b]Eating difficulties*	-0.28 (0.72)	1.07 (1.32)	0.63 (1.22)	-0.12 (0.78)	0.85 (1.28)
[e]Eating difficulties: Pressure to eat*	-0.30 (0.84)	0.85 (0.97)	0.83 (0.94)	-0.28 (0.87)	0.78 (1.03)
[c]Language*	-0.06 (0.87)	0.19 (1.26)	0.04 (0.99)	0.05 (1.10)	0.19 (1.33)
[c]Motor skills*	-0.01 (0.98)	0.20 (1.13)	0.05 (1.02)	0.09 (1.07)	0.18 (1.16)
[f]Social communication*	-0.07 (0.91)	0.25 (1.19)	0.09 (1.04)	0.06 (1.05)	0.22 (1.20)
[f]Restricted and repetitive*	-0.11 (0.96)	0.17 (1.02)	0.13 (1.01)	0.00 (0.99)	0.11 (1.00)
[b]Externalizing*	-0.12 (0.95)	0.30 (1.08)	0.20 (1.02)	0.01 (1.01)	0.20 (1.05)
[b]Internalizing*	-0.20 (0.86)	0.67 (1.19)	0.39 (1.10)	0.01 (0.96)	0.58 (1.19)
[b]Attention/hyperactivity*	-0.11 (0.96)	0.25 (1.09)	0.19 (1.02)	0.01 (1.01)	0.18 (1.04)
[b]Aggressive/uncooperative	-0.08 (0.96)	0.24 (1.10)	0.16 (1.04)	0.01 (1.01)	0.12 (1.06)
5 years					
[b]Eating difficulties*	-0.20 (0.83)	1.04 (1.32)	0.29 (1.11)	0.23 (1.09)	0.87 (1.32)
[c]Language*	-0.03 (0.92)	0.20 (1.34)	0.05 (0.99)	0.07 (1.11)	0.22 (1.40)
[g]Motor skills*	-0.03 (0.95)	0.35 (1.39)	0.02 (0.97)	0.12 (1.16)	0.32 (1.44)
[h]Social communication*	-0.08 (0.94)	0.27 (1.19)	0.05 (0.99)	0.13 (1.08)	0.20 (1.16)
[h]Restricted and repetitive*	-0.09 (0.95)	0.24 (1.10)	0.08 (1.05)	0.04 (1.02)	0.19 (1.08)
[b]Externalizing*	-0.09 (0.94)	0.28 (1.11)	0.12 (1.02)	0.07 (1.05)	0.22 (1.08)
[b]Internalizing*	-0.14 (0.89)	0.59 (1.21)	0.16 (1.05)	0.18 (1.10)	0.44 (1.18)

© 2025 Bjørndal LD et al. *JAMA Pediatrics*.

[b]Attention/hyperactivity*	-0.08 (0.95)	0.25 (1.11)	0.14 (1.02)	0.07 (1.04)	0.21 (1.08)
[b]Aggressive/uncooperative	-0.07 (0.95)	0.20 (1.11)	0.08 (1.02)	0.04 (1.06)	0.12 (1.05)
8 years					
[i]Eating difficulties: Satiety responsiveness*	-0.21 (0.86)	1.09 (1.20)	0.02 (0.92)	0.77 (1.09)	0.77 (1.17)
[i]Eating difficulties: Fussiness*	-0.18 (0.86)	1.16 (1.18)	0.11 (0.90)	0.51 (1.27)	0.53 (1.16)
[i]Eating difficulties: Food responsiveness*	0.04 (1.02)	-0.27 (0.84)	-0.03 (0.96)	-0.18 (0.91)	-0.24 (0.79)
[i]Eating difficulties: Emotional overeating*	0.01 (1.00)	-0.14 (0.97)	0.02 (1.00)	-0.14 (0.96)	-0.10 (0.95)
[i]Eating difficulties: Emotional undereating*	-0.08 (0.93)	0.27 (1.23)	0.00 (0.94)	0.40 (1.31)	0.16 (1.13)
[a]Delayed/atypical language development, n (%)*	1,604 (6.61)	218 (10.25)	492 (7.77)	300 (10.00)	280 (12.37)
[a]Delayed psychomotor development, n (%)*	317 (1.31)	90 (4.23)	106 (1.67)	97 (3.23)	125 (5.52)
[f]Social communication*	-0.06 (0.96)	0.22 (1.10)	0.07 (1.03)	0.07 (1.07)	0.14 (1.12)
[f]Restricted and repetitive*	-0.07 (0.89)	0.36 (1.40)	0.05 (1.05)	0.14 (1.18)	0.31 (1.42)
[j]Emotional: Depressive*	-0.08 (0.92)	0.33 (1.25)	0.05 (1.01)	0.19 (1.15)	0.22 (1.16)
[k]Emotional: Anxiety*	-0.06 (0.94)	0.25 (1.23)	0.02 (0.98)	0.15 (1.12)	0.19 (1.17)
[l]Attention/hyperactivity*	-0.09 (0.91)	0.41 (1.24)	0.08 (1.02)	0.21 (1.21)	0.31 (1.25)
[l]Aggressive/uncooperative*	-0.01 (0.96)	0.09 (1.11)	0.03 (1.02)	0.06 (1.15)	0.07 (1.11)
14 years, mother-reported					
[a]Delayed/atypical language development, n (%)*	414 (3.37)	74 (7.20)	115 (3.79)	92 (6.09)	74 (6.89)
[m]Social communication*(Prosocial behavior)	-0.03 (0.97)	0.27 (1.17)	0.07 (1.03)	0.09 (1.05)	0.15 (1.08)
[j]Emotional: Depressive*	-0.05 (0.96)	0.15 (1.16)	0.00 (0.97)	0.09 (1.08)	0.16 (1.09)
[k]Emotional: Anxiety*	-0.05 (0.93)	0.19 (1.12)	-0.01 (0.96)	0.10 (1.14)	0.18 (1.16)
[l]Attention/hyperactivity*	-0.09 (0.91)	0.30 (1.21)	0.01 (1.01)	0.11 (1.09)	0.27 (1.22)
[l]Aggressive/uncooperative*	-0.05 (0.95)	0.17 (1.13)	0.07 (1.05)	0.10 (1.07)	0.17 (1.12)
14 years, child-reported					
[n]Eating difficulties: Restraint	-0.02 (0.99)	-0.08 (0.94)	-0.05 (0.95)	-0.04 (0.96)	-0.12 (0.87)
[n]Eating difficulties: Shape	-0.02 (0.99)	-0.01 (0.96)	-0.05 (0.97)	-0.02 (0.98)	-0.05 (0.99)
[m]Social communication (Prosocial behavior)*	-0.01 (0.99)	0.12 (1.06)	0.01 (1.01)	0.02 (0.95)	0.01 (1.01)
[o]Emotional: Anxiety and depressive	-0.04 (0.98)	0.05 (1.02)	-0.05 (0.98)	0.04 (1.00)	0.08 (1.03)
[j]Emotional: Depressive	-0.04 (0.99)	0.04 (1.01)	-0.04 (0.98)	0.02 (0.98)	0.02 (0.99)
[k]Emotional: Anxiety	-0.04 (0.98)	0.02 (1.01)	-0.05 (0.97)	0.06 (1.02)	0.07 (1.00)

© 2025 Bjørndal LD et al. *JAMA Pediatrics*.

[l]Aggressive/uncooperative	-0.02 (0.97)	0.02 (1.01)	0.02 (1.10)	-0.01 (0.90)	0.01 (0.95)

Note. [a]MoBa item, [b]CBCL, [c]ASQ, [d]M-CHAT, [e]CFQ, [f]SCQ, [g]CDI, [h]CAST, [i]CEBQ, [j]SMFQ, [k]SCARED, [l]RS-DBD, [m]SDQ, [n]EDE-Q, [o]SCL-10. Unless otherwise stated, all values reported are mean scores with standard deviations in parenthesis. Each scale was standardized (i.e., with mean = 0, standard deviation = 1), including all available data in the total MoBa sample. Characteristics for which independent sample t-tests or two-proportion *z*-tests yielded statistically significant differences ($\alpha = .05$) with p-values corrected for the FDR are indicated by *, when comparing children with ARFI-Broad Persistent and no ARFI-Broad.

© 2025 Bjørndal LD et al. *JAMA Pediatrics*.

eTable 6.

Test Statistics Comparing Developmental Characteristics in Children With ARFI-Persistent and no ARFI-Broad.

Age	Characteristics	t/χ^2	p (FDR)
—	Male sex, n (%)	13.52	.013
0.5 years	Breastfeeding problems, n (%)	22.95	< .001
1.5 years	Eating difficulties	-34.48	< .001
	Language	-6.90	< .001
	Motor skills	-8.04	< .001
	Social communication	-7.96	< .001
	Restricted and repetitive	-7.87	< .001
	Externalizing	-12.66	< .001
	Internalizing	-22.23	< .001
	Attention/hyperactivity	-11.11	< .001
	Aggressive/uncooperative	-9.75	< .001
3 years	Eating difficulties	-46.70	< .001
	Eating difficulties: Pressure to eat	-52.66	< .001
	Language	-8.96	< .001
	Motor skills	-8.17	< .001
	Social communication	-12.27	< .001
	Restricted and repetitive	-12.39	< .001
	Externalizing	-17.12	< .001
	Internalizing	-32.82	< .001
	Attention/hyperactivity	-14.79	< .001
	Aggressive/uncooperative	-13.12	< .001
5 years	Eating difficulties	-35.52	< .001
	Language	-6.45	< .001
	Motor skills	-10.17	< .001
	Social communication	-6.56	< .001
	Restricted and repetitive	-6.66	< .001
	Externalizing	-12.47	< .001
	Internalizing	-22.50	< .001
	Attention/hyperactivity	-11.15	< .001
	Aggressive/uncooperative	-9.12	< .001
8 years	Eating difficulties: Satiety responsiveness	-49.08	< .001
	Eating difficulties: Fussiness	-51.15	< .001
	Eating difficulties: Food responsiveness	15.93	< .001
	Eating difficulties: Emotional overeating	7.07	< .001

© 2025 Bjørndal LD et al. *JAMA Pediatrics*.

	Eating difficulties: Emotional undereating	-12.91	< .001
	Delayed/atypical language development, n (%)	38.87	< .001
	Delayed psychomotor development, n (%)	108.42	< .001
	Social communication	-11.33	< .001
	Restricted and repetitive	-14.14	< .001
	Emotional: Depressive	-14.66	< .001
	Emotional: Anxiety	-11.40	< .001
	Attention/hyperactivity	-18.27	< .001
	Aggressive/uncooperative	-4.12	< .001
14 years, mother-reported	Delayed/atypical language development, n (%)	38.35	< .001
	Social communication (Prosocial behavior)	-7.75	< .001
	Emotional: Depressive	-5.37	< .001
	Emotional: Anxiety	-6.74	< .001
	Attention/hyperactivity	-9.98	< .001
	Aggressive/uncooperative	-6.11	< .001
14 years, child-reported	Eating difficulties: Restraint	1.82	0.969
	Eating difficulties: Shape	-0.28	1.000
	Social communication (Prosocial behavior)	-3.46	0.031
	Emotional: Anxiety and depressive	-2.57	0.286
	Emotional: Depressive	-2.11	0.653
	Emotional: Anxiety	-1.71	0.979
	Aggressive/uncooperative	-1.20	1.000

Note. t-values are reported for independent samples t-tests and χ^2-values for tests of proportions (these are further indicated by 'n (%)' being included for each characteristic of relevance). For the t-tests, a negative t-value ($t < 0$) indicates that children classified with ARFI-Persistent have higher scores on the given characteristic than children with no ARFI-Broad. Conversely, positive t-values ($t > 0$) indicate that children classified with ARFI-Persistent have lower scores on the given characteristic (e.g., for eating difficulties food responsiveness and emotional overeating at 8 years).

© 2025 Bjørndal LD et al. *JAMA Pediatrics*.

eTable 7.

Maternal Sociodemographic Characteristics at MoBa Q1 for Children With no ARFI-Broad and Children With ARFI-Broad Persistent.

**No ARFI-Broad**		**ARFI-Broad Persistent**	
Characteristic	n (%) or M (SD)	Characteristic	n (%) or M (SD)
Age, years	30.82 (4.27)	*Age, years*	30.53 (4.61)
Marital status		*Marital status*	
Married	12,593 (51.86%)	Married	1,043 (48.99%)
Divorced or separated	41 (0.17%)	Divorced or separated	5 (0.23%)
Cohabiting	10,952 (45.10%)	Cohabiting	977 (45.89%)
Single	299 (1.23%)	Single	56 (2.63%)
Widowed and other	187 (0.77%)	Widowed and other	18 (0.85%)
Education level (completed)		*Education level (completed)*	
No higher education	5,739 (23.63%)	No higher education	668 (31.38%)
Higher education	17,400 (71.66%)	Higher education	1,329 (62.42%)
Household income		*Household income*	
No income	328 (1.35%)	No income	47 (2.21%)
< 150.000 NOK	2,812 (11.58%)	< 150.000 NOK	302 (14.19%)
151.000 - 299.999 NOK	10,180 (41.92%)	151.000 - 299.999 NOK	848 (39.83%)
300.000-499.999 NOK	8,943 (36.83%)	300.000-499.999 NOK	753 (35.37%)
> 500.000 NOK	1,240 (5.11%)	> 500.000 NOK	104 (4.88%)

Note. Percentages may not add to 100 due to missing data.

© 2025 Bjørndal LD et al. *JAMA Pediatrics*.

eTable 8.

Characteristics Among Children With ARFI-Clinical.

**Characteristics**	**Any ARFI-Broad and clinical indicator**	**ARFI-Broad Transient and clinical indicator**	**ARFI-Broad Emergent and clinical indicator**	**ARFI-Broad Persistent and clinical indicator**
	(n = 2,265)	(n = 1,157)	(n = 484)	(n = 624)
Male sex (registry), n (%)	1161 (51.26)	592 (51.17)	232 (47.93)	337 (54.01)
0.5 years				
Breastfeeding problems, n (%)	145 (6.40)	76 (6.57)	30 (6.20)	39 (6.25)
1.5 years				
Eating difficulties	0.69 (1.26)	0.67 (1.22)	0.25 (1.15)	1.07 (1.29)
Language	0.18 (1.12)	0.13 (1.08)	0.16 (1.12)	0.28 (1.19)
Motor skills	0.27 (1.40)	0.20 (1.30)	0.22 (1.29)	0.44 (1.61)
Social communication	0.18 (1.29)	0.15 (1.17)	0.11 (1.20)	0.28 (1.54)
Restricted and repetitive	0.12 (1.04)	0.09 (0.99)	0.05 (0.97)	0.25 (1.15)
Externalizing	0.11 (1.03)	0.11 (1.00)	0.06 (1.07)	0.15 (1.04)
Internalizing	0.48 (1.20)	0.42 (1.14)	0.27 (1.14)	0.74 (1.30)
Attention/hyperactivity	0.13 (1.06)	0.13 (1.05)	0.09 (1.04)	0.16 (1.10)
Aggressive/uncooperative	0.03 (1.02)	0.02 (0.99)	0.02 (1.04)	0.07 (1.04)
3 years				
Eating difficulties	0.85 (1.28)	0.92 (1.23)	-0.03 (0.80)	1.40 (1.32)
Eating difficulties: Pressure to eat	0.78 (1.03)	1.03 (0.92)	-0.12 (0.90)	1.01 (0.94)
Language	0.19 (1.33)	0.13 (1.15)	0.12 (1.30)	0.36 (1.63)
Motor skills	0.18 (1.16)	0.13 (1.07)	0.14 (1.14)	0.31 (1.30)
Social communication	0.22 (1.20)	0.15 (1.06)	0.16 (1.23)	0.39 (1.40)
Restricted and repetitive	0.11 (1.00)	0.12 (0.97)	0.01 (0.99)	0.19 (1.06)
Externalizing	0.20 (1.05)	0.25 (1.04)	0.01 (1.00)	0.27 (1.09)
Internalizing	0.58 (1.19)	0.58 (1.18)	0.17 (1.01)	0.87 (1.26)
Attention/hyperactivity	0.18 (1.04)	0.23 (1.02)	0.00 (0.98)	0.22 (1.10)
Aggressive/uncooperative	0.12 (1.06)	0.17 (1.06)	-0.03 (1.02)	0.17 (1.08)
5 years				
Eating difficulties	0.87 (1.32)	0.66 (1.24)	0.58 (1.18)	1.47 (1.36)
Language	0.22 (1.40)	0.15 (1.18)	0.13 (1.23)	0.43 (1.82)
Motor skills	0.32 (1.44)	0.15 (1.19)	0.28 (1.30)	0.67 (1.85)
Social communication	0.20 (1.16)	0.12 (1.14)	0.22 (1.15)	0.33 (1.18)
Restricted and repetitive	0.19 (1.08)	0.12 (1.02)	0.23 (1.19)	0.28 (1.10)
Externalizing	0.22 (1.08)	0.21 (1.08)	0.12 (1.08)	0.31 (1.09)
Internalizing	0.44 (1.18)	0.31 (1.12)	0.35 (1.17)	0.75 (1.25)
Attention/hyperactivity	0.21 (1.08)	0.21 (1.07)	0.13 (1.05)	0.26 (1.12)
Aggressive/uncooperative	0.12 (1.05)	0.12 (1.05)	0.06 (1.05)	0.18 (1.06)
8 years				

© 2025 Bjørndal LD et al. *JAMA Pediatrics*.

Eating difficulties: Satiety responsiveness	0.77 (1.17)	0.24 (0.94)	1.17 (1.02)	1.44 (1.18)
Eating difficulties: Fussiness	0.53 (1.16)	0.17 (0.91)	0.56 (1.26)	1.19 (1.20)
Eating difficulties: Food responsiveness	-0.24 (0.79)	-0.14 (0.86)	-0.34 (0.69)	-0.36 (0.71)
Eating difficulties: Emotional overeating	-0.10 (0.95)	0.00 (0.98)	-0.24 (0.89)	-0.19 (0.92)
Eating difficulties: Emotional undereating	0.16 (1.13)	0.01 (0.96)	0.29 (1.25)	0.32 (1.26)
Delayed/atypical language development, n (%)	280 (12.37)	128 (11.06)	63 (13.02)	89 (14.29)
Delayed psychomotor development, n (%)	125 (5.52)	45 (3.89)	26 (5.37)	54 (8.67)
Social communication	0.14 (1.12)	0.07 (1.03)	0.17 (1.21)	0.26 (1.19)
Restricted and repetitive	0.31 (1.42)	0.13 (1.18)	0.36 (1.49)	0.61 (1.68)
Emotional: Depressive	0.22 (1.16)	0.11 (1.07)	0.31 (1.22)	0.35 (1.25)
Emotional: Anxiety	0.19 (1.17)	0.07 (1.03)	0.23 (1.23)	0.38 (1.33)
Attention/hyperactivity	0.31 (1.25)	0.16 (1.13)	0.39 (1.33)	0.54 (1.38)
Aggressive/uncooperative	0.07 (1.11)	0.06 (1.05)	0.13 (1.31)	0.03 (1.07)
14 years, mother-reported				
Delayed/atypical language development, n (%)	74 (6.89)	26 (4.78)	21 (9.05)	27 (9.06)
Social communication: Prosocial behavior	0.15 (1.08)	0.05 (0.98)	0.11 (1.10)	0.34 (1.21)
Emotional: Depressive	0.16 (1.09)	0.09 (0.98)	0.23 (1.16)	0.24 (1.22)
Emotional: Anxiety	0.18 (1.16)	0.08 (1.01)	0.24 (1.43)	0.31 (1.17)
Attention/hyperactivity	0.27 (1.22)	0.14 (1.12)	0.28 (1.20)	0.48 (1.37)
Aggressive/uncooperative	0.17 (1.12)	0.13 (1.08)	0.23 (1.11)	0.20 (1.19)
14 years, child-reported				
Eating difficulties: Restraint	-0.12 (0.87)	-0.13 (0.85)	-0.11 (0.88)	-0.13 (0.92)
Eating difficulties: Shape	-0.05 (0.99)	-0.09 (0.96)	0.02 (1.03)	-0.02 (1.00)
Social communication: Prosocial behavior	0.01 (1.01)	-0.01 (1.03)	-0.01 (0.86)	0.06 (1.10)
Emotional: Anxiety and depressive	0.08 (1.03)	-0.01 (1.01)	0.24 (1.01)	0.12 (1.06)
Emotional: Depressive	0.02 (0.99)	-0.05 (0.96)	0.19 (1.05)	0.03 (1.00)
Emotional: Anxiety	0.07 (1.00)	0.01 (0.97)	0.20 (1.05)	0.06 (1.02)
Aggressive/uncooperative	0.01 (0.95)	-0.01 (0.96)	0.19 (1.03)	-0.02 (0.86)

© 2025 Bjørndal LD et al. *JAMA Pediatrics*.

eTable 9.
Lifetime Diagnoses Among Children With ARFI-Clinical.

Diagnoses	ARFI-Transient and clinical indicator	ARFI-Emergent and clinical indicator	ARFI-Persistent and clinical indicator
	(n = 1,157)	(n = 484)	(n = 624)
Intellectual disability (F70-F79)	16 (1.38%)	9 (1.86%)	22 (3.53%)
Global developmental delay (F83)	12 (1.04%)	9 (1.86%)	10 (1.60%)
OCD (F42)	21 (1.82%)	14 (2.89%)	9 (1.44%)
Autism (F84.0, F84.1, F84.5, F84.8, F84.9)	37 (3.20%)	26 (5.37%)	59 (9.46%)
ADHD (F90)	97 (8.38%)	57 (11.78%)	77 (12.34%)
Anorexia nervosa (F50.0 and F50.1)	11 (0.95%)	7 (1.45%)	7 (1.12%)
Other eating disorders (F50.2, F50.3, F50.4, F50.5)	< 5	< 5	< 5
Neurological: Epilepsy (G40)	30 (2.59%)	22 (4.55%)	21 (3.37%)
Gastrointestinal: Crohn's disease (K50), ulcerative colitis (K51), irritable bowel syndrome (K58), celiac disease (K90.0)	76 (6.57%)	34 (7.02%)	44 (7.05%)

© 2025 Bjørndal LD et al. *JAMA Pediatrics*.

eTable 10.

SNV-Heritability Estimates, Sensitivity Analyses With More Stringent Inclusion Criteria.

Phenotype	SNV-h2	SE	p
ARFI-Broad: 3 years	.078	.026	.002
ARFI-Broad: 8 years	.085	.043	.025
ARFI-Broad: 3 or 8 years (Ever)	.024	.024	<.001
ARFI-Broad: 3 and 8 years (Persistent)	.052	.087	.274
ARFI-Clinical: 3 or 8 years (Ever)	.145	.068	.017
ARFI-Clinical: 3 and 8 years (Persistent)	.134	.182	.230

Note. For these sensitivity analyses, we additionally excluded cases with diagnoses of general medical conditions that could plausibly explain the dysregulated eating pattern (Table S2).

eTable 11.

Results From Gene-Based Association Analyses.

Phenotype	N GWS Genes	Gene(s)	Chr	Start	End	N SNVs	Z	p
ARFI-Clinical 3 or 8 years	1	ADCY3	2	25042038	25142602	49	5.4171	3.0287e-08

© 2025 Bjørndal LD et al. *JAMA Pediatrics*.

eTable 12.

Genetic Correlation Estimates.

	ARFI-Broad								ARFI-Clinical			
	3 years		8 years		3 or 8 years (Ever)		3 and 8 years (Persistent)		3 or 8 years (Ever)		3 and 8 years (Persistent)	
Phenotype	rg (SE)	p	rg (SE)	p	rg (SE)	p	rg (SE)	p	rg (SE)	p	rg (SE)	p
OCD	.11 (.16)	.49	.00 (.16)	.98	.07 (.14)	.62	—		.14 (.19)	.44	—	
Autism	.03 (.11)	.80	.19 (.10)	.07	.05 (.09)	.62	.24 (.73)	.74	.11 (.12)	.40	—	
ADHD	.03 (.09)	.68	.07 (.09)	.43	.04 (.08)	.57	.07 (.25)	.78	.21 (.10)	<.05	.28 (.42)	.51
AN	.08 (.09)	.38	.11 (.09)	.23	.07 (.08)	.38	.39 (.56)	.49	-.01 (.11)	.96	.09 (.31)	.78
Anxiety	.20 (.25)	.41	.17 (.28)	.55	.19 (.22)	.40	.42 (1.71)	.81	.41 (.28)	.14	-.18 (.89)	.84
Epilepsy (all)	-.04 (.12)	.77	-.01 (.11)	.91	-.00 (.11)	.99	-.22 (.27)	.41	-.02 (.11)	.87	-.38 (.38)	.32
Epilepsy (eu)	-.06 (.12)	.64	-.00 (.11)	.99	-.01 (.11)	.90	-.22 (.27)	.42	-.04 (.12)	.76	-.37 (.38)	.33
Cognitive ability	-.29 (.08)	<.001	-.02 (.07)	.73	-.21 (.07)	.001	-.21 (.53)	.69	-.24 (.08)	<.05	—	
Educational attainment	-.37 (.07)	<.001	-.15 (.07)	.03	-.33 (.07)	<.001	-.30 (.51)	.56	-.25 (.08)	<.05	—	
Birth weight	-.15 (.15)	.34	-.00 (.10)	.98	-.09 (.11)	.39	—		-.20 (.09)	<.05	-.28 (.46)	.55
BMI	-.17 (.06)	<.05	-.21 (.06)	<.001	-.18 (.06)	<.05	-.45 (.69)	.51	-.31 (.08)	<.001	—	
Childhood BMI	-.55 (.12)	<.001	-.70 (.15)	<.001	-.56 (.11)	<.001	—		-.94 (.18)	<.001	—	
Childhood BMI 2025	-.63 (.11)	<.001	-.68 (.14)	<.001	-.63 (.10)	<.001	—		-1.00 (.18)	<.001	—	
IBS	.13 (.10)	.21	.07 (.10)	.49	.13 (.09)	.15	-.04 (.27)	.87	.14 (.10)	.19	—	
IBD	.10 (.08)	.20	.19 (.09)	<.05	.10 (.06)	.10	—		.15 (.09)	.11	—	
Ulcerative colitis	.25 (.11)	<.05	.26 (.11)	<.05	.23 (.09)	<.05	—		.15 (.11)	.17	—	
Crohn's disease	.01 (.08)	.88	.11 (.08)	.18	.03 (.07)	.68	—		.14 (.10)	.14	—	
Celiac disease	—		.46 (.26)	.08	.43 (.37)	.25	—		.49 (.24)	.04	.10 (.24)	.69
F-acquired	-.43 (.10)	<.001	-.39 (.10)	<.001	-.42 (.09)	<.001	-.83 (1.06)	.43	-.36 (.11)	<.001	—	
F-Caffeinated sweet drinks	.39 (.11)	<.001	.41 (.13)	<.05	.36 (.10)	<.001	1.13 (1.74)	.52	.51 (.13)	<.001	—	
F-Highly palatable	.18 (.12)	.12	.03 (.09)	.75	.11 (.10)	.26	.30 (.47)	.52	.15 (.11)	.17	—	
F-Low caloric	-.35 (.10)	<.001	-.39 (.11)	<.001	-.34 (.09)	<.001	-1.16 (2.16)	.59	-.38 (.11)	<.001	—	
F-Savoury	-.31 (.10)	.001	-.36 (.10)	<.001	-.31 (.08)	<.001	-.97 (1.59)	.54	-.22 (.10)	<.05	—	
Leptin	-.18 (.17)	.29	-.27 (.16)	.09	-.21 (.14)	.15	-.52 (1.10)	.64	-.09 (.18)	.59	—	
Leptin adj. BMI	-.12 (.17)	.47	.16 (.17)	.36	-.05 (.15)	.76	.34 (.67)	.61	.33 (.21)	.11	—	

Note. Non-estimable genetic correlation estimates are indicated by —. *F* represents 'food-liking' phenotypes.

© 2025 Bjørndal LD et al. *JAMA Pediatrics*.

eTable 13.

Genetic Correlation Estimates, Sensitivity Analyses With More Stringent Inclusion Criteria.

	ARFI-Broad								ARFI-Clinical			
	3 years		8 years		Ever		Persistent		Ever		Persistent	
Phenotype	rg (SE)	p	rg (SE)	p	rg (SE)	p	rg (SE)	p	rg (SE)	p	rg (SE)	p
OCD	.16 (.17)	.35	-.03 (.20)	.90	.11 (.15)	.45	.28 (.48)	.56	.18 (.21)	.40	-.49 (.53)	.36
Autism	.02 (.11)	.89	.16 (.13)	.22	.01 (.10)	.91	.24 (.34)	.49	.09 (.13)	.51	.38 (.35)	.27
ADHD	.01 (.09)	.92	.02 (.10)	.84	.01 (.08)	.91	-.01 (.17)	.96	.19 (.10)	.06	.11 (.16)	.48
AN	.10 (.10)	.32	.20 (.12)	.10	.10 (.09)	.23	.36 (.31)	.24	.06 (.13)	.66	.12 (.20)	.54
Anxiety	.25 (.26)	.34	.21 (.31)	.50	.22 (.23)	.35	.53 (.87)	.54	.40 (.31)	.19	-.23 (.52)	.66
Epilepsy (all)	-.01 (.13)	.91	.02 (.13)	.88	.03 (.12)	.78	-.16 (.22)	.46	.03 (.12)	.80	-.26 (.22)	.23
Epilepsy (eu)	-.05 (.13)	.73	.03 (.13)	.83	.02 (.12)	.89	-.17 (.23)	.45	.01 (.13)	.95	-.25 (.22)	.25
Cognitive ability	-.28 (.08)	<.001	.02 (.08)	.84	-.19 (.07)	<.05	-.20 (.26)	.46	-.26 (.09)	<.05	-.05 (.15)	.72
Educational attainment	-.40 (.08)	<.001	-.15 (.09)	.10	-.34 (.07)	<.001	-.24 (.27)	.37	-.28 (.09)	<.05	-.22 (.19)	.24
Birth weight	-.13 (.13)	.34	.04 (.12)	.75	-.08 (.10)	.43	—		-.23 (.10)	<.05	-.11 (.19)	.56
BMI	-.18 (.07)	<.05	-.24 (.08)	<.05	-.19 (.06)	<.05	-.36 (.27)	.19	-.36 (.11)	<.001	—	
Childhood BMI	-.55 (.13)	<.001	-.81 (.23)	<.001	-.56 (.12)	<.001	—		-1.04 (.24)	<.001	—	
Childhood BMI 2025	-.63 (.12)	<.001	-.80 (.22)	<.001	-.64 (.11)	<.001	-1.19 (1.18)	.31	-1.05 (.23)	<.001	-1.12 (.77)	.15
IBS	.08 (.11)	.47	.01 (.12)	.92	.08 (.10)	.41	-.08 (.23)	.72	.08 (.12)	.50	-.07 (.23)	.75
IBD	.15 (.09)	.08	.26 (.12)	<.05	.14 (.07)	.05	.60 (.65)	.36	.19 (.11)	.10	.41 (.34)	.23
Ulcerative colitis	.30 (.12)	<.05	.37 (.15)	<.05	.28 (.10)	<.05	.69 (.73)	.34	.20 (.14)	.16	.45 (.39)	.25
Crohn's disease	.05 (.08)	.54	.13 (.11)	.21	.04 (.07)	.56	.33 (.41)	.42	.18 (.11)	.10	.24 (.25)	.34
Celiac disease	—		.48 (.72)	.50	.27 (.39)	.48	—		.52 (.48)	.28	.02 (.17)	.93
F-acquired	-.40 (.10)	<.001	-.41 (.13)	<.05	-.39 (.09)	<.001	-.70 (.59)	.23	-.40 (.12)	<.05	-.46 (.33)	.17
F-Caffeinated sweet drinks	.35 (.11)	<.05	.38 (.16)	<.05	.30 (.10)	<.05	.93 (.92)	.31	.51 (.15)	<.001	.92 (.64)	.15
F-Highly palatable	.20 (.12)	.09	.03 (.11)	.79	.14 (.10)	.19	.22 (.26)	.40	.14 (.11)	.20	.38 (.30)	.21
F-Low caloric	-.32 (.11)	<.05	-.43 (.15)	<.05	-.32 (.10)	<.05	-.94 (1.01)	.35	-.42 (.13)	<.05	-.66 (.47)	.16
F-Savoury	-.26 (.10)	<.05	-.41 (.13)	<.05	-.27 (.08)	<.05	-.85 (.84)	.31	-.19 (.10)	.06	-.36 (.29)	.22
Leptin	-.17 (.18)	.34	-.23 (.19)	.23	-.19 (.16)	.21	-.28 (.50)	.57	-.02 (.20)	.92	-.30 (.42)	.48
Leptin adj. BMI	-.16 (.18)	.38	-.19 (.21)	.35	-.09 (.16)	.59	.26 (.39)	.51	.37 (.22)	.10	.12 (.41)	.78

Note. For these sensitivity analyses, we additionally excluded cases with diagnoses of general medical conditions that could plausibly explain the dysregulated eating pattern (Table S2). Non-estimable genetic correlation estimates are indicated by —. *F* represents 'food-liking' phenotypes.

© 2025 Bjørndal LD et al. *JAMA Pediatrics*.

eFigure 2.
Manhattan Plot: ARFI-Broad Emergent (8 Years), Cases = 4,430, Controls = 26,107.

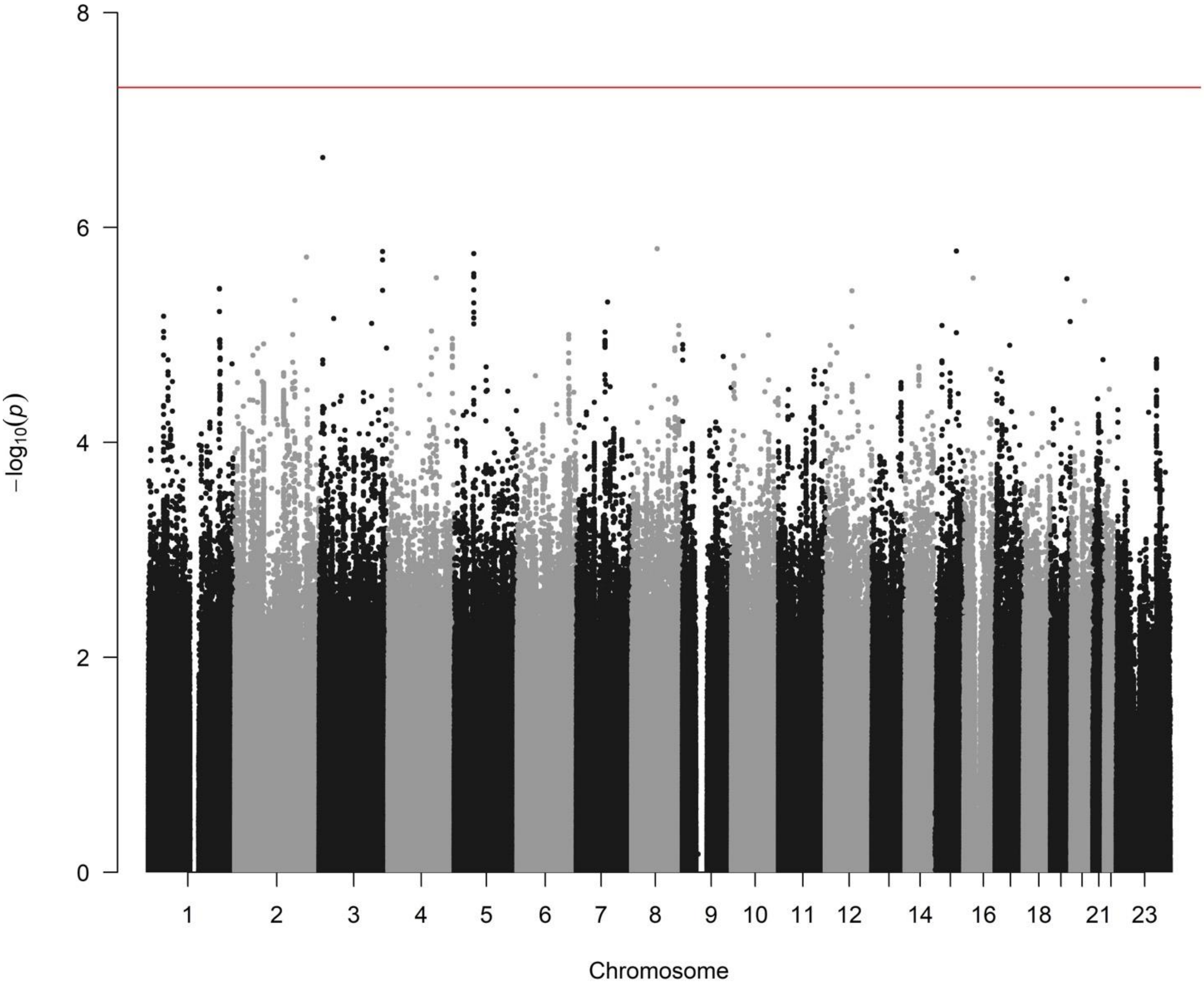

© 2025 Bjørndal LD et al. *JAMA Pediatrics*.

eFigure 3.
Manhattan Plot: ARFI-Broad at 3 or 8 Years, Cases = 13,128, Controls = 26,107.

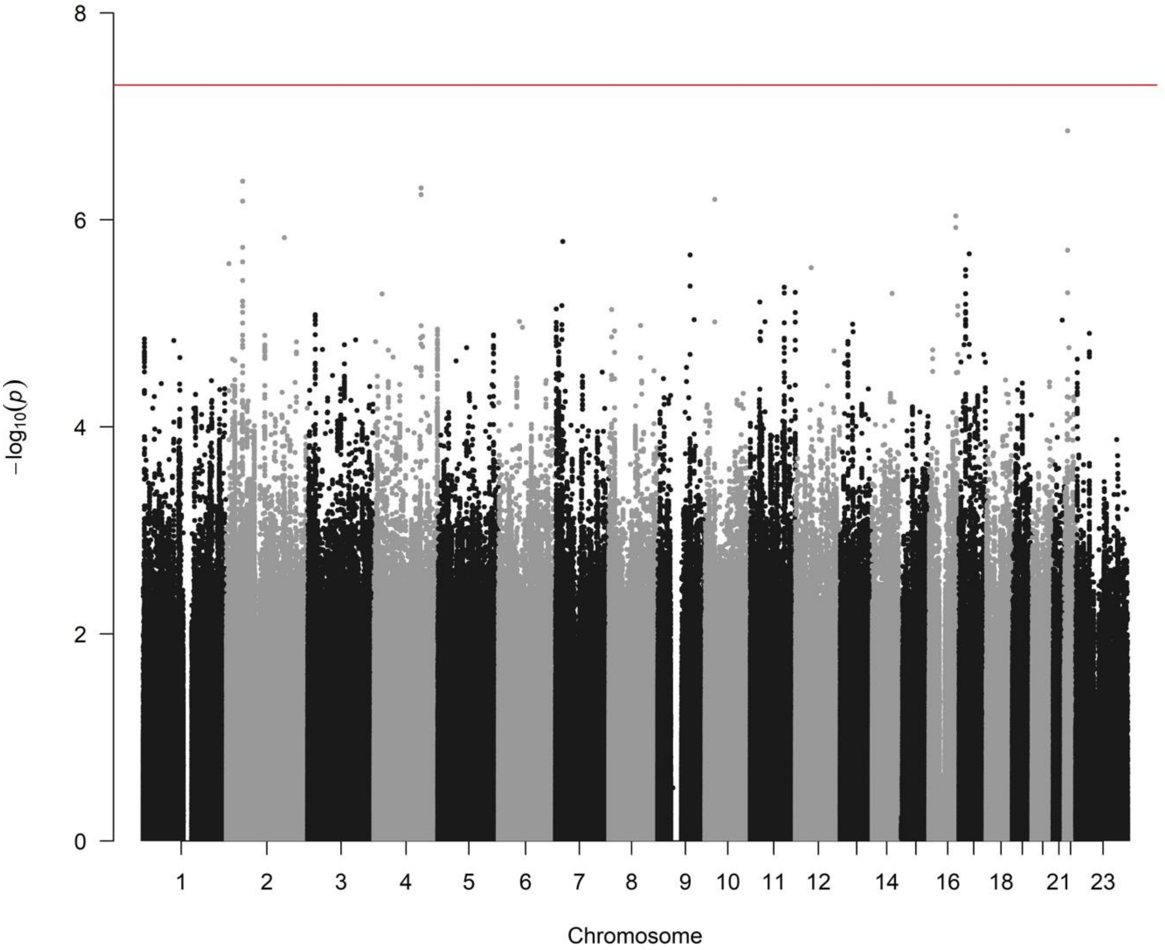

© 2025 Bjørndal LD et al. *JAMA Pediatrics*.

eFigure 4.
Manhattan Plot: ARFI-Broad Persistent, Cases = 1,521, Controls = 26,107.

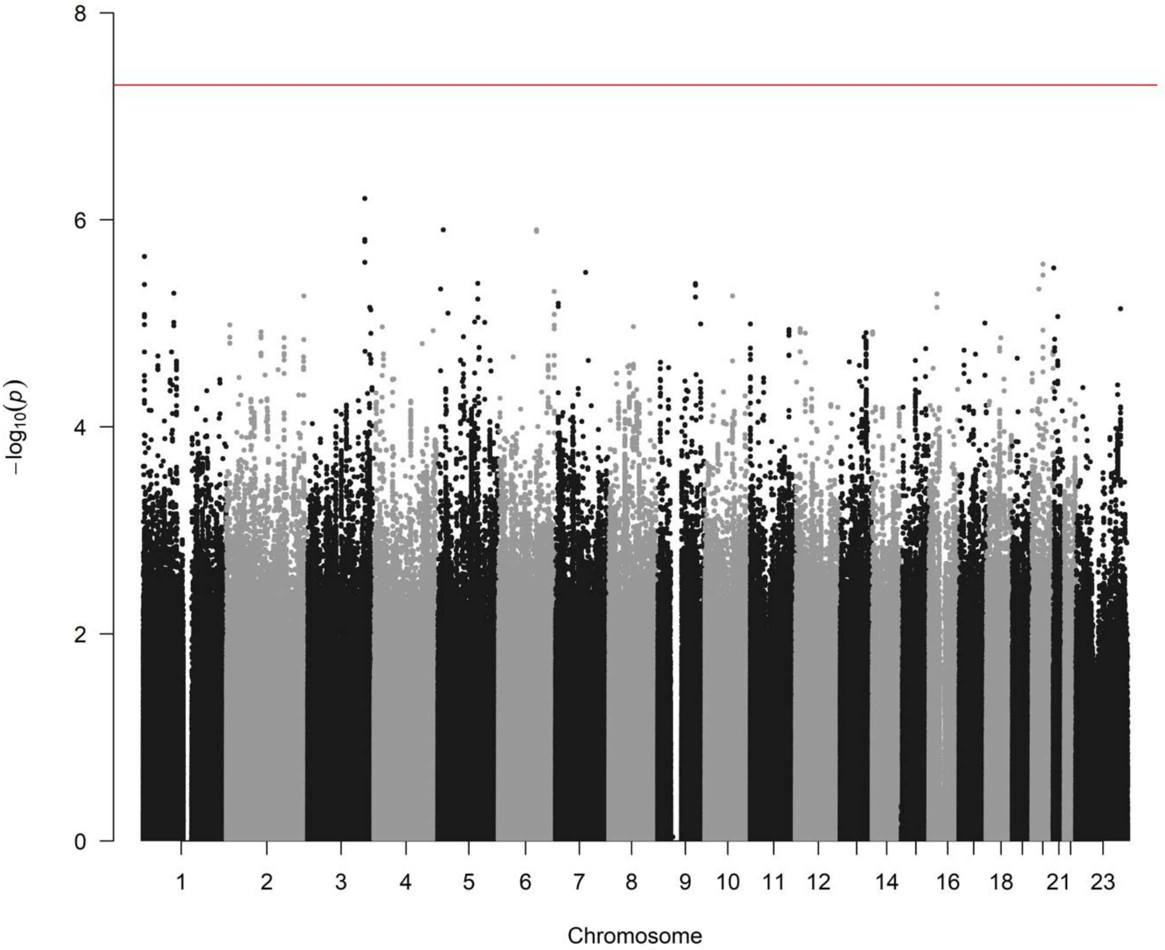

© 2025 Bjørndal LD et al. *JAMA Pediatrics*.

eFigure 5.
Manhattan Plot: ARFI-Clinical Persistent, Cases = 452, Controls = 26,107.

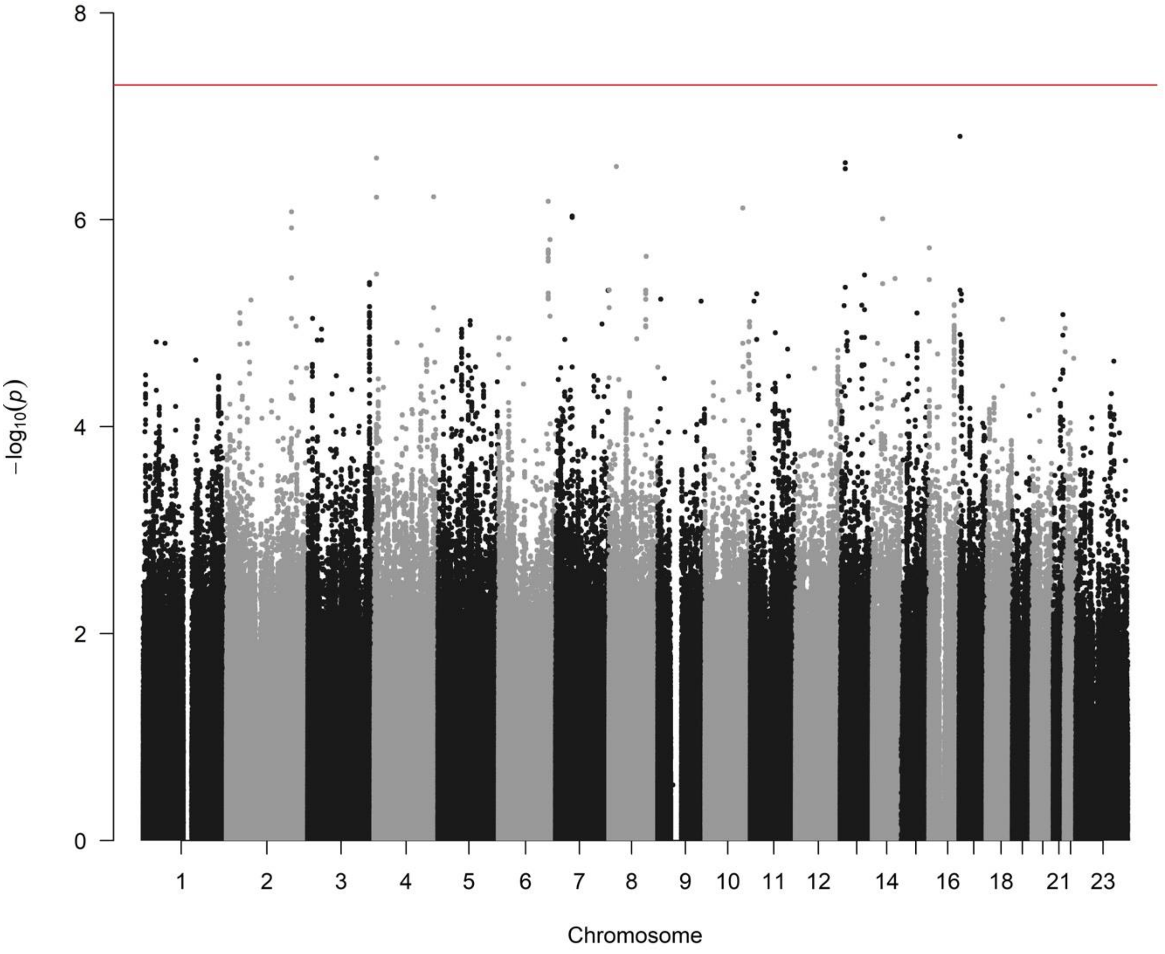

© 2025 Bjørndal LD et al. *JAMA Pediatrics*.

eFigure 6.
Manhattan Plot: ARFI-Broad Transient (3 Years), Sensitivity Analysis, Cases = 8,994, Controls = 26,107

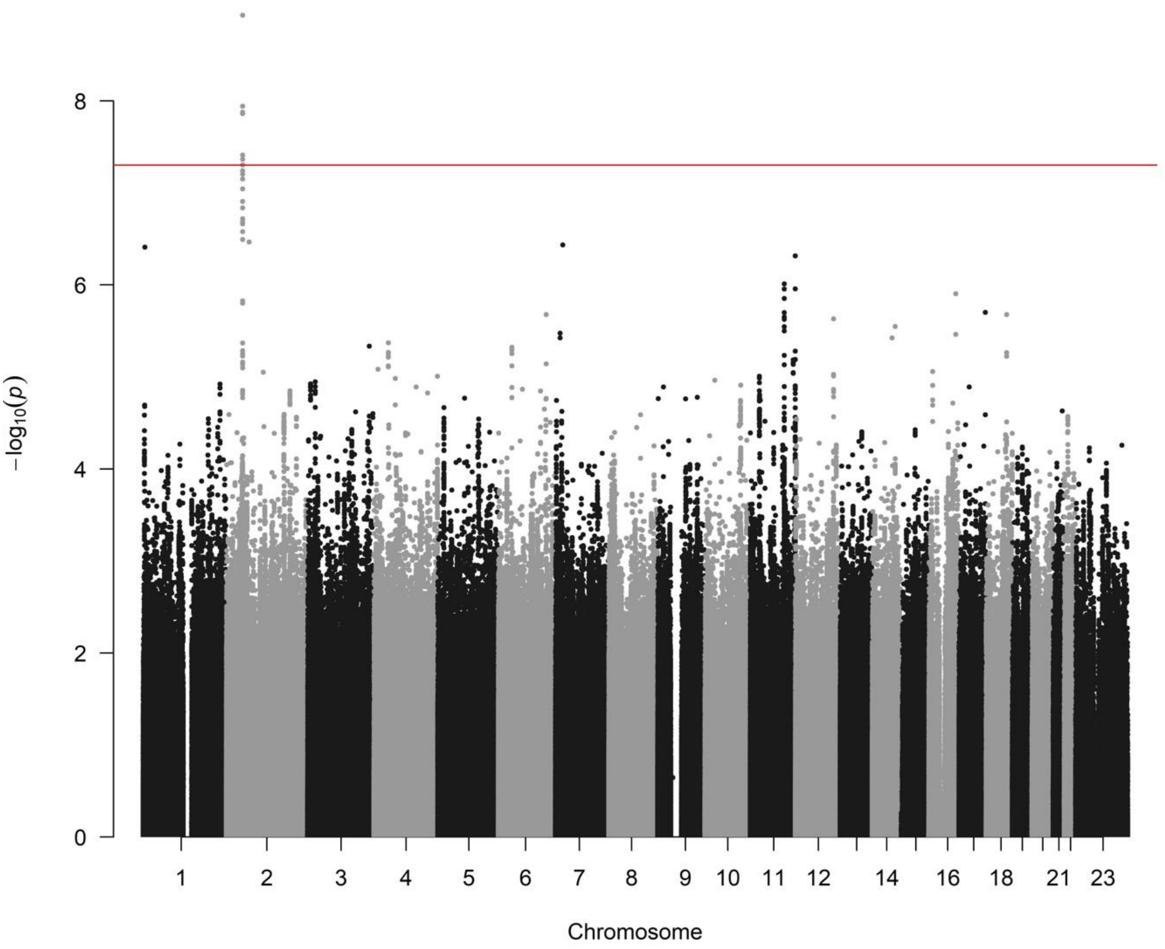

Note. For these sensitivity analyses, we additionally excluded cases with diagnoses of general medical conditions that could plausibly explain the dysregulated eating pattern (Table S2).

© 2025 Bjørndal LD et al. *JAMA Pediatrics*.

eFigure 7.
Manhattan Plot: ARFI-Broad Emergent (8 Years), Sensitivity Analysis, Cases = 3,856, Controls = 26,107.

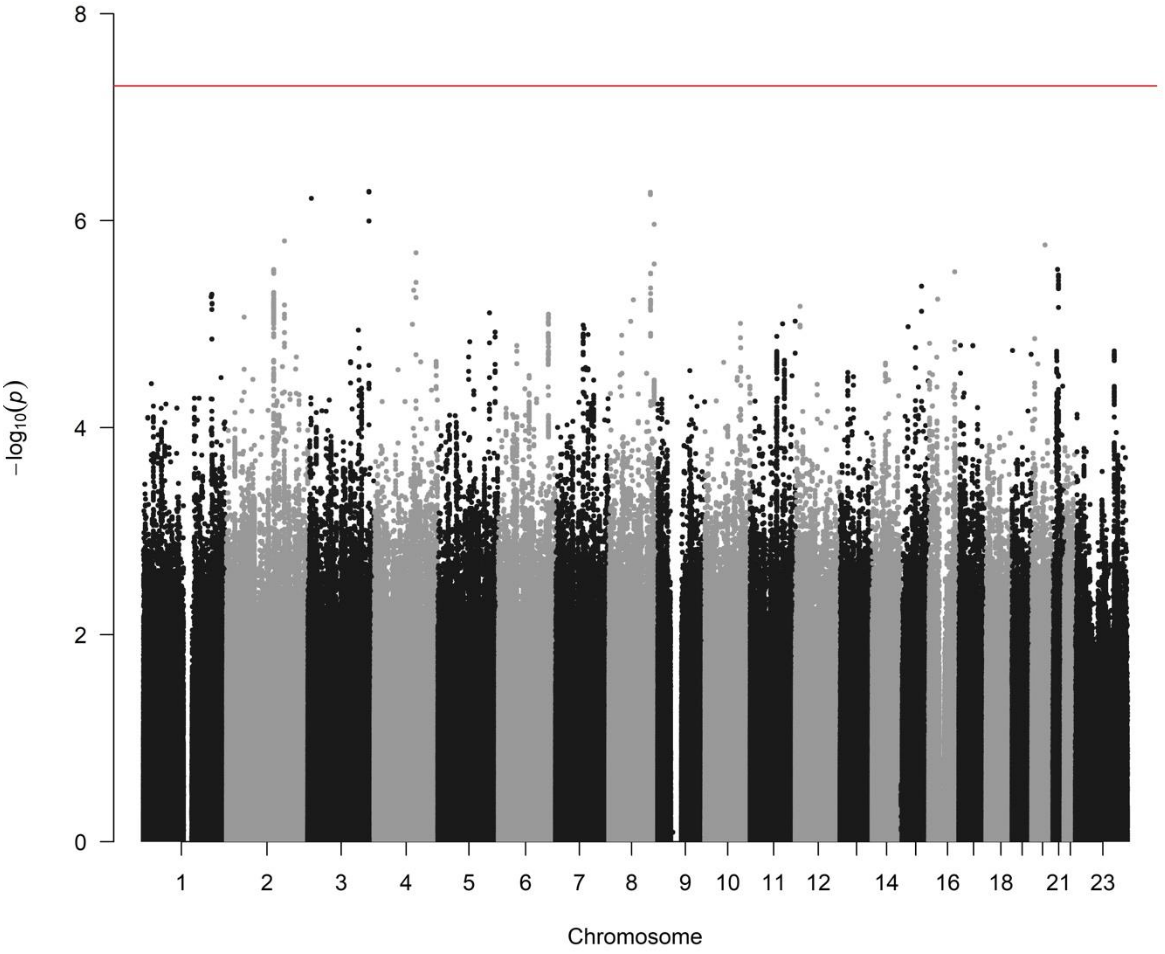

Note. For these sensitivity analyses, we additionally excluded cases with diagnoses of general medical conditions that could plausibly explain the dysregulated eating pattern (Table S2).

© 2025 Bjørndal LD et al. *JAMA Pediatrics*.

eFigure 8.

Manhattan Plot: ARFI-Broad at 3 or 8 Years, Sensitivity Analyses, Cases = 11,548, Controls = 26,107.

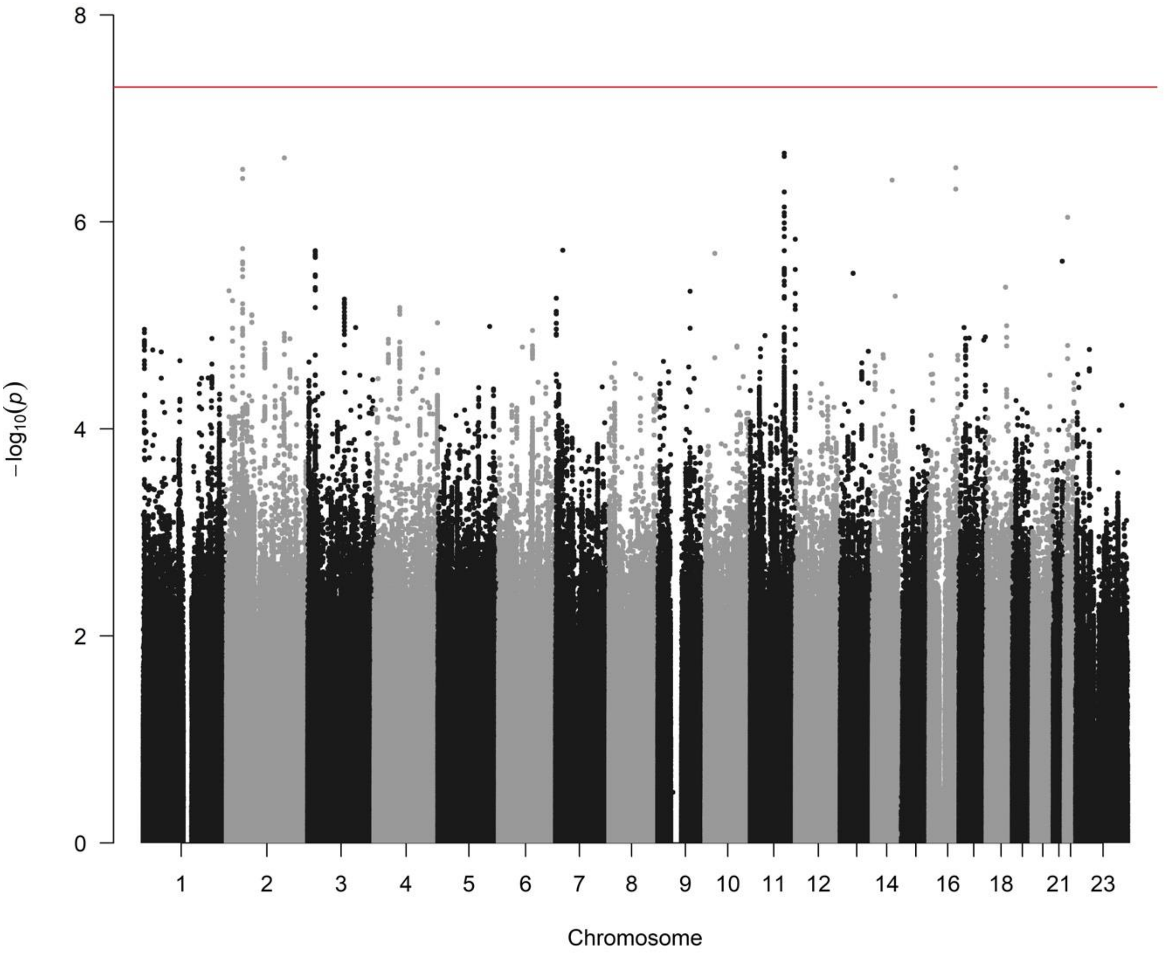

Note. For these sensitivity analyses, we additionally excluded cases with diagnoses of general medical conditions that could plausibly explain the dysregulated eating pattern (Table S2).

© 2025 Bjørndal LD et al. *JAMA Pediatrics*.

eFigure 9.

Manhattan Plot: ARFI-Broad Persistent, Sensitivity Analysis, Cases = 1,302, Controls = 26,107.

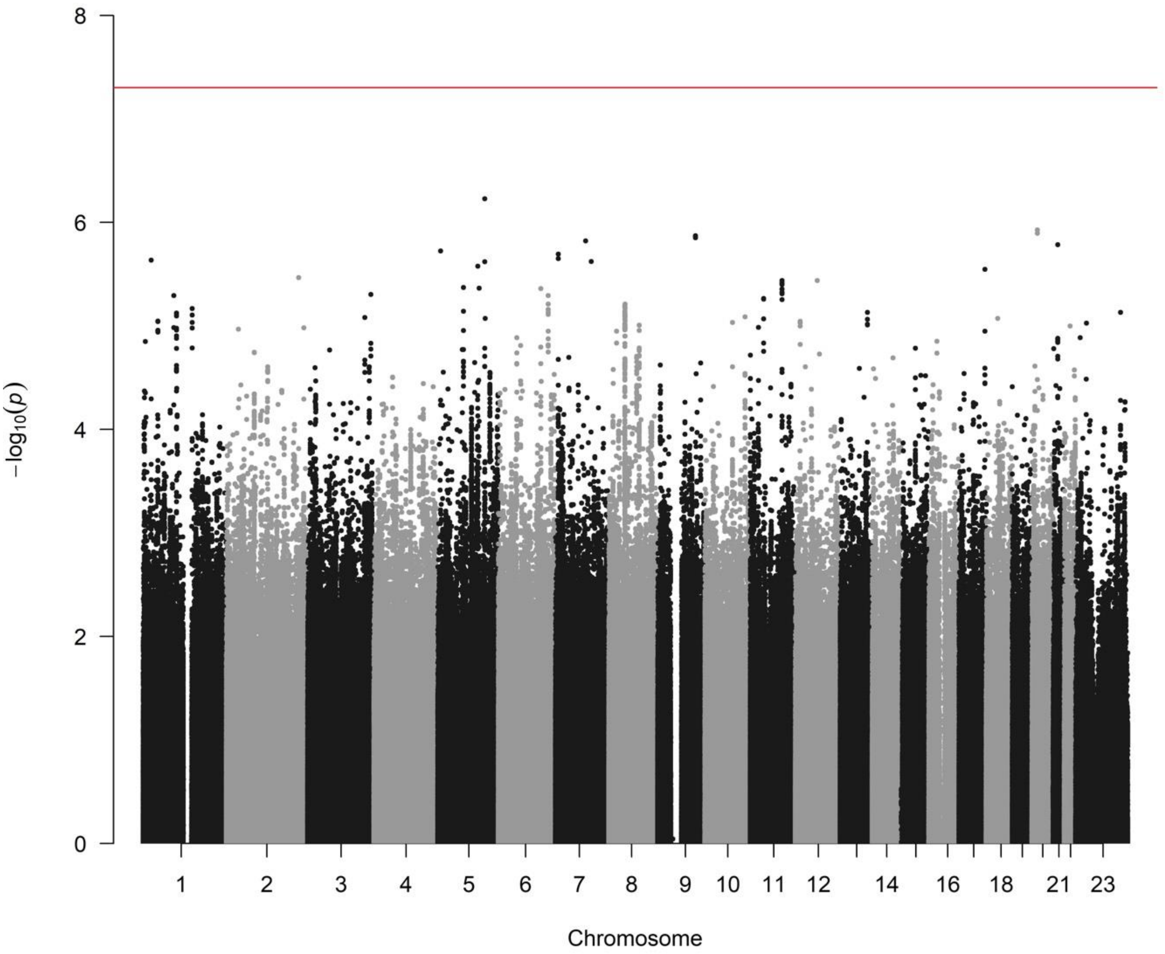

Note. For these sensitivity analyses, we additionally excluded cases with diagnoses of general medical conditions that could plausibly explain the dysregulated eating pattern (Table S2).

© 2025 Bjørndal LD et al. *JAMA Pediatrics*.

eFigure 10.

Manhattan Plot: ARFI-Clinical Persistent, Sensitivity Analysis, Cases = 363, Controls = 26,107.

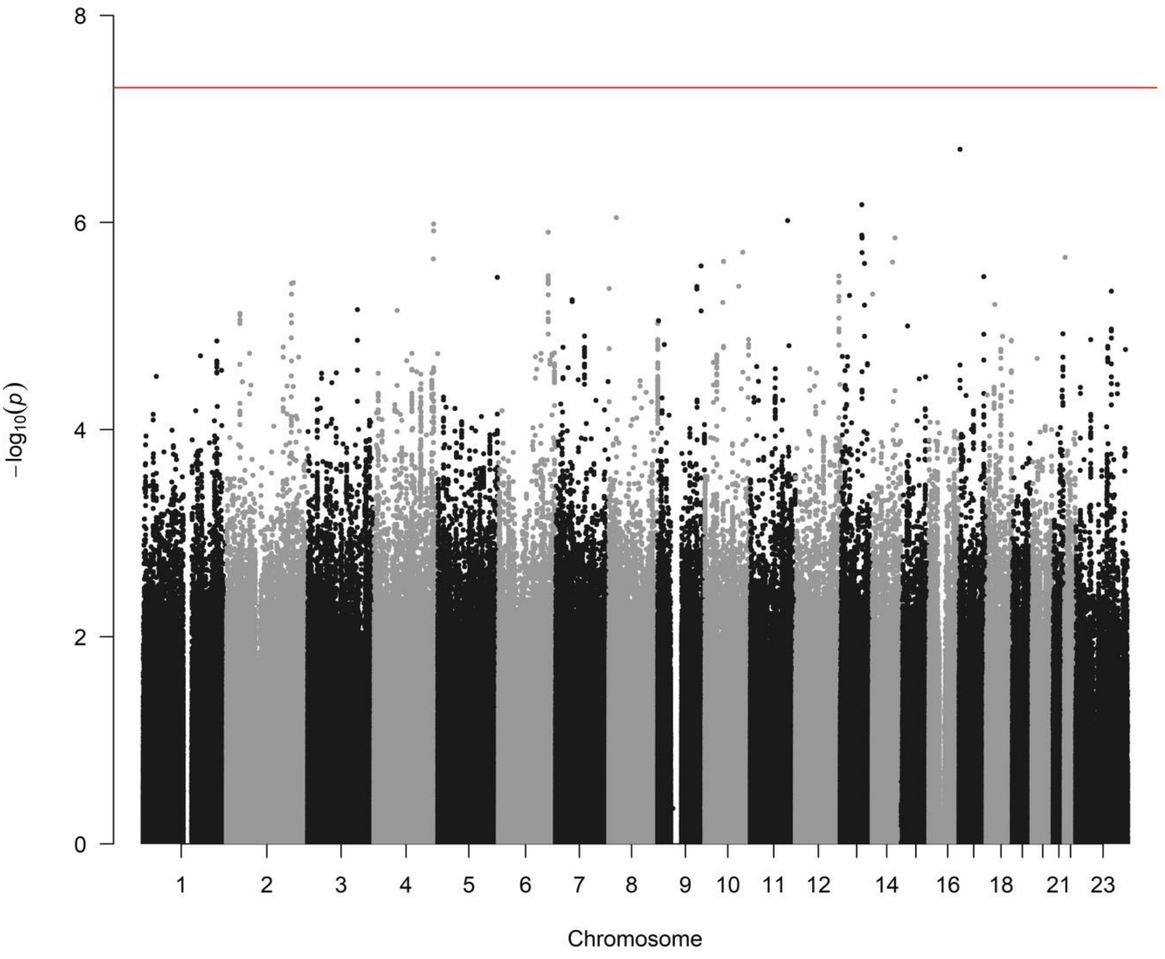

Note. For these sensitivity analyses, we additionally excluded cases with diagnoses of general medical conditions that could plausibly explain the dysregulated eating pattern (Table S2).

© 2025 Bjørndal LD et al. *JAMA Pediatrics*.

eFigure 11.

Manhattan Plot: ARFI-Clinical at 3 or 8 Years, Sensitivity Analysis, Cases = 1,893, Controls = 26,107.

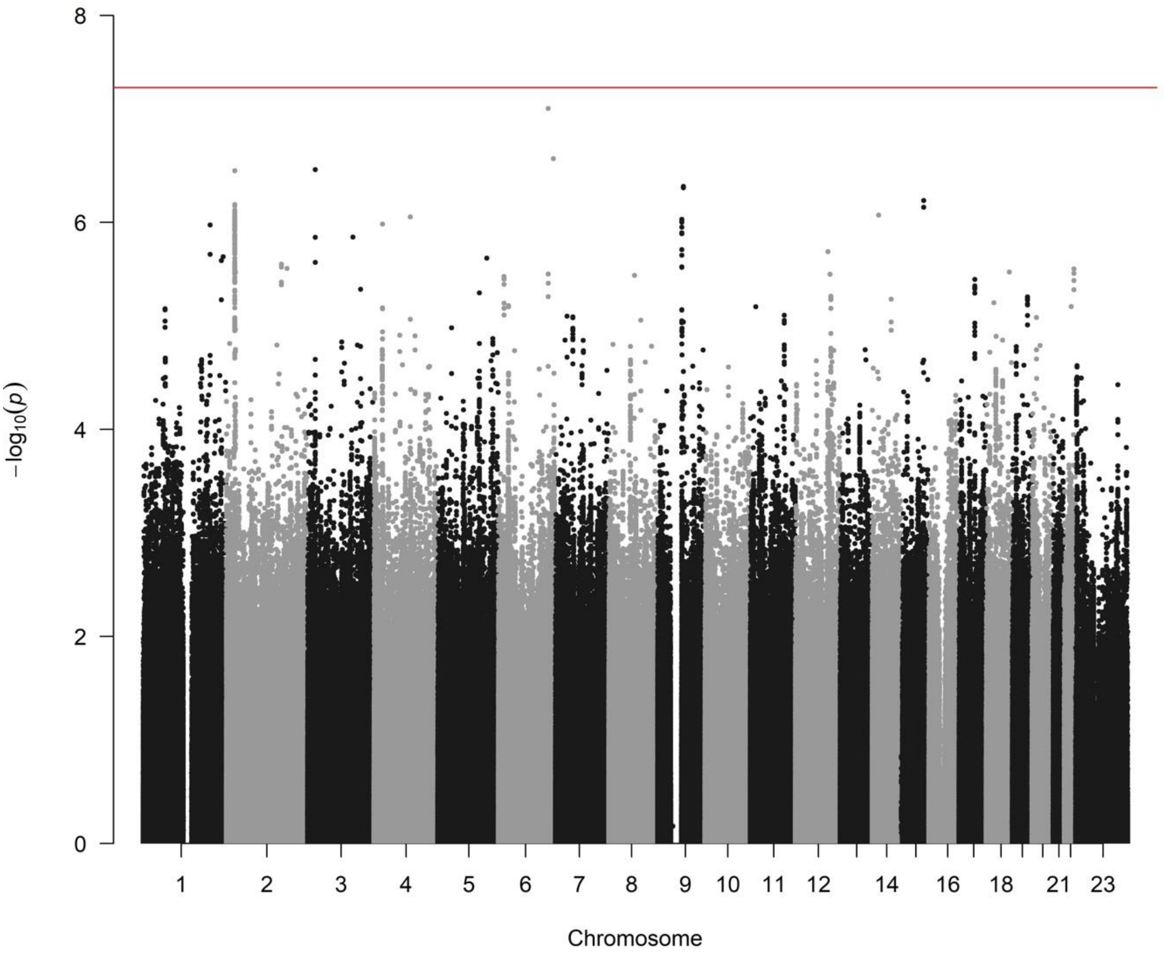

Note. For these sensitivity analyses, we additionally excluded cases with diagnoses of general medical conditions that could plausibly explain the dysregulated eating pattern (Table S2).

© 2025 Bjørndal LD et al. *JAMA Pediatrics*.

eFigure 12.

Regional Plot: ARFI-Broad Transient (3 Years).

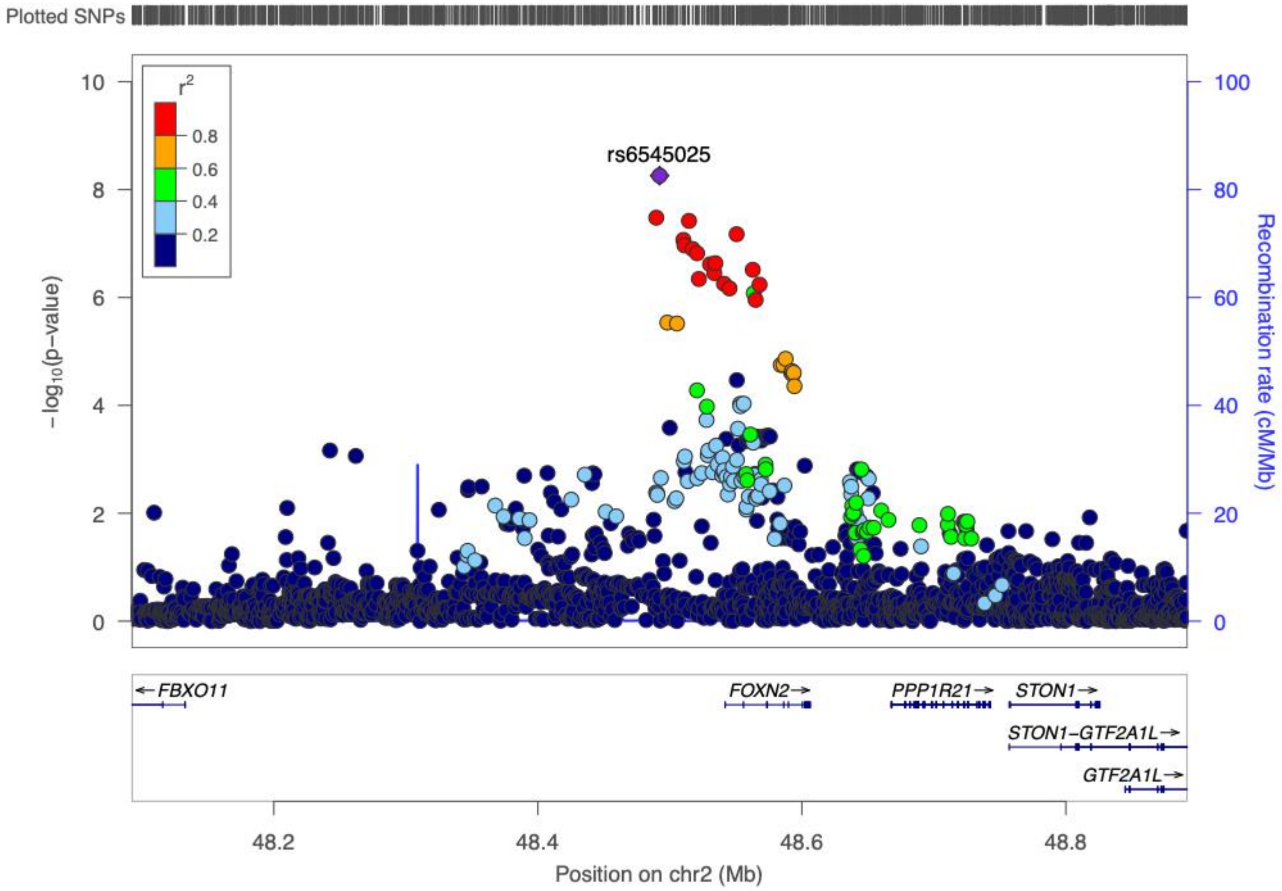

© 2025 Bjørndal LD et al. *JAMA Pediatrics*.

eFigure 13.

Regional Plot: ARFI-Clinical at 3 or 8 Years.

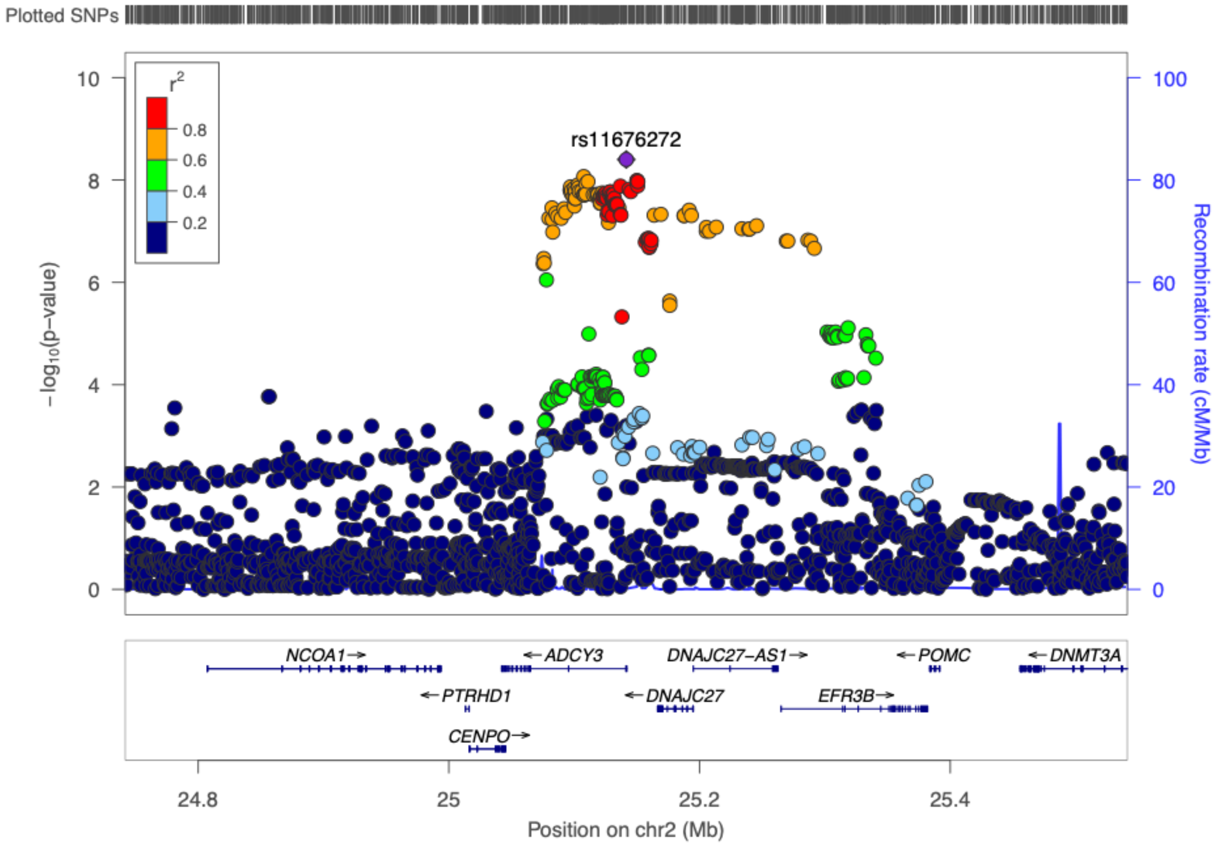

© 2025 Bjørndal LD et al. *JAMA Pediatrics*.

eReferences

1. Questionnaires from MoBa. Norwegian Institute of Public Health. 2024. Accessed March 31, 2025. https://www.fhi.no/en/ch/studies/moba/for-forskere-artikler/questionnaires-from-moba/
2. International Obsessive Compulsive Disorder Foundation Genetics Collaborative (IOCDF-GC), OCD Collaborative Genetics Association Studies (OCGAS). Revealing the complex genetic architecture of obsessive-compulsive disorder using meta-analysis. *Mol Psychiatry*. 2018;23(5):1181-1188. doi:10.1038/mp.2017.154
3. Grove J, Ripke S, Als TD, et al. Identification of common genetic risk variants for autism spectrum disorder. *Nat Genet*. 2019;51(3):431-444. doi:10.1038/s41588-019-0344-8
4. Demontis D, Walters GB, Athanasiadis G, et al. Genome-wide analyses of ADHD identify 27 risk loci, refine the genetic architecture and implicate several cognitive domains. *Nat Genet*. 2023;55(2):198-208. doi:10.1038/s41588-022-01285-8
5. Watson HJ, Yilmaz Z, Thornton LM, et al. Genome-wide association study identifies eight risk loci and implicates metabo-psychiatric origins for anorexia nervosa. *Nat Genet*. 2019;51(8):1207-1214. doi:10.1038/s41588-019-0439-2
6. Otowa T, Hek K, Lee M, et al. Meta-analysis of genome-wide association studies of anxiety disorders. *Mol Psychiatry*. 2016;21(10):1391-1399. doi:10.1038/mp.2015.197
7. International League Against Epilepsy Consortium on Complex Epilepsies. GWAS meta-analysis of over 29,000 people with epilepsy identifies 26 risk loci and subtype-specific genetic architecture. *Nat Genet*. 2023;55(9):1471-1482. doi:10.1038/s41588-023-01485-w
8. Savage JE, Jansen PR, Stringer S, et al. Genome-wide association meta-analysis in 269,867 individuals identifies new genetic and functional links to intelligence. *Nat Genet*. 2018;50(7):912-919. doi:10.1038/s41588-018-0152-6
9. Lee JJ, Wedow R, Okbay A, et al. Gene discovery and polygenic prediction from a 1.1-million-person GWAS of educational attainment. *Nat Genet*. 2018;50(8):1112-1121. doi:10.1038/s41588-018-0147-3
10. Warrington NM, Beaumont RN, Horikoshi M, et al. Maternal and fetal genetic effects on birth weight and their relevance to cardio-metabolic risk factors. *Nat Genet*. 2019;51(5):804-814. doi:10.1038/s41588-019-0403-1
11. Yengo L, Sidorenko J, Kemper KE, et al. Meta-analysis of genome-wide association studies for height and body mass index in ∼700000 individuals of European ancestry. *Hum Mol Genet*. 2018;27(20):3641-3649. doi:10.1093/hmg/ddy271
12. Vogelezang S, Bradfield JP, Ahluwalia TS, et al. Novel loci for childhood body mass index and shared heritability with adult cardiometabolic traits. *PLOS Genetics*. 2020;16(10):e1008718. doi:10.1371/journal.pgen.1008718
13. Downie CG, Shrestha P, Okello S, et al. Trans-ancestry genome-wide association study of childhood body mass index identifies novel loci and age-specific effects. *Human Genetics and Genomics Advances*. 2025;6(2):100411. doi:10.1016/j.xhgg.2025.100411
14. Eijsbouts C, Zheng T, Kennedy NA, et al. Genome-wide analysis of 53,400 people with irritable bowel syndrome highlights shared genetic pathways with mood and anxiety disorders. *Nat Genet*. 2021;53(11):1543-1552. doi:10.1038/s41588-021-00950-8
15. de Lange KM, Moutsianas L, Lee JC, et al. Genome-wide association study implicates immune activation of multiple integrin genes in inflammatory bowel disease. *Nat Genet*. 2017;49(2):256-261. doi:10.1038/ng.3760
16. Trynka G, Hunt KA, Bockett NA, et al. Dense genotyping identifies and localizes multiple common and rare variant association signals in celiac disease. *Nat Genet*. 2011;43(12):1193-1201. doi:10.1038/ng.998
17. May-Wilson S, Matoba N, Wade KH, et al. Large-scale GWAS of food liking reveals

© 2025 Bjørndal LD et al. *JAMA Pediatrics*.

genetic determinants and genetic correlations with distinct neurophysiological traits. *Nat Commun*. 2022;13(1):2743. doi:10.1038/s41467-022-30187-w
18. Kilpeläinen TO, Carli JFM, Skowronski AA, et al. Genome-wide meta-analysis uncovers novel loci influencing circulating leptin levels. *Nat Commun*. 2016;7(1):10494. doi:10.1038/ncomms10494
19. Willroth EC, Atherton OE. Best Laid Plans: A Guide to Reporting Preregistration Deviations. *Advances in Methods and Practices in Psychological Science*. 2024;7(1):25152459231213802. doi:10.1177/25152459231213802

© 2025 Bjørndal LD et al. *JAMA Pediatrics*.
