# Supplementary material for: Prevalence, Characteristics, and Genetic Architecture of Avoidant/Restrictive Food Intake Phenotypes
Source: JAMA Pediatr. 2025 Nov 24;180(1):45–55. doi: 10.1001/jamapediatrics.2025.4786 (PMC12645403; doi:10.1001/jamapediatrics.2025.4786)
Supplement: Supplement 2. — Data Sharing Statement. [file jamapediatr-e254786-s002.pdf]

Data Sharing Statement

Bjørndal. Prevalence, Characteristics, and Genetic Architecture of Avoidant/Restrictive Food Intake Phenotypes. *JAMA Pediatr*. Published November 24, 2025.
doi:10.1001/jamapediatrics.2025.4786

Data
Data available: No

Additional Information
Explanation for why data not available: Data from the Norwegian Mother, Father and Child Cohort Study and the Medical Birth Registry of Norway used in this study are managed by the national health register holders in Norway (Norwegian Institute of Public Health) and can be made available to researchers, provided approval from the Regional Committees for Medical and Health Research Ethics (REC), compliance with the EU General Data Protection Regulation (GDPR) and approval from the data owners. The consent given by the participants does not open for storage of data on an individual level in repositories or journals. Researchers who want access to data sets for replication should apply through helsedata.no. Access to data sets requires approval from The Regional Committee for Medical and Health Research Ethics in Norway.
